# Circular RNA profiling identifies circ102049 as a key regulator of colorectal liver metastasis

**DOI:** 10.1002/1878-0261.12840

**Published:** 2020-12-29

**Authors:** Qiaoming Zhi, Daiwei Wan, Rui Ren, Zhihua Xu, Xiaobo Guo, Ye Han, Fei Liu, Ye Xu, Lei Qin, Yilin Wang

**Affiliations:** ^1^ Department of Colorectal Surgery Fudan University Shanghai Cancer Center China; ^2^ Department of General Surgery The First Affiliated Hospital of Soochow University Suzhou China; ^3^ Department of General Surgery The Second Affiliated Hospital of Soochow University Suzhou China; ^4^ Department of Gastrointestinal Surgery Provincial Hospital Affiliated to Shandong University Jinan China; ^5^ Department of Gastroenterology The First Affiliated Hospital of Soochow University Suzhou China; ^6^ Department of Hepatic Surgery Fudan University Shanghai Cancer Center Shanghai Medical College China

**Keywords:** colorectal liver metastasis, DGCR8, FRAS1, Hsa_circ_102049, miR‐192‐3p, miR‐761

## Abstract

Circular RNA (circRNA) plays an essential role in the development and progression of various cancers. However, the functions and mechanisms of circRNA in colorectal liver metastasis have not been fully elucidated. We performed circRNA microarray analysis to screen differentially expressed circRNA in the pathology of colorectal liver metastasis. Quantitative real‐time PCR was used to detect the expression of hsa_circ_102049 (circ102049) in colorectal cancer (CRC) samples. CRC cells were transfected with circ102049 overexpression vector or small interfering (si)RNA to assess the effects of circ102049 *in vitro*. Bioinformatics analysis, fluorescence in situ hybridization, RNA immunoprecipitation, RNA pull‐down and luciferase reporter assays were conducted to confirm the relationship of circ102049, miR‐761, miR‐192‐3p and FRAS1. The mechanism by which circ102049 recruits and distributes DGCR8 protein in the cytoplasm was also investigated. We found that circ102049 was highly expressed in primary CRC tumors with liver metastasis and closely correlated with the prognosis of patients with CRC. Circ102049 significantly enhanced the adhesion, migration and invasion abilities of CRC cells, and promoted CRC progression via a micro (mi)R‐761/miR‐192‐3p‐FRAS1‐dependent mechanism. Notably, due to the distribution of DGCR8 protein, circ102049 may also indirectly reduce the levels of mature miR‐761 and miR‐192‐3p in the cytoplasm. In addition, the role of circ102049 in promoting colorectal liver metastasis was confirmed *in vivo*. Our findings provide new evidence that circ102049 may be a potential prognostic factor in CRC, and that the circ102049‐miR‐761/miR‐192‐3p–FRAS1 axis may be an anti‐metastatic target for CRC patients.

## Graphical abstract

Here, we identify circ102049 as a regulator of metastatic colorectal cancer (CRC). High circ102049 levels were correlated with poor clinical outcomes in patients with CRC and promoted liver metastasis in an FRAS1‐dependent manner. Circ102049 regulated the levels of the FRAS1‐targeting miR‐761 and miR‐192‐3p both directly (acting as a miRNA sponge) and indirectly (modifying the subcellular localization of the RNA binding protein DGCR8).

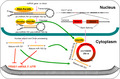

**Abbreviations**
CCK‐8: cell counting kit‐8
CeRNA: competing endogenous RNA
ChIRP: chromatin isolation by RNA purification
circRNA: circular RNA
CRC: colorectal cancer
FISH: fluorescence in situ hybridization
GSEA: gene set enrichment analysis
ncRNA: non‐coding RNA
OS: overall survival
pre‐miRNA: precursor miRNA
pri‐miRNA: primary miRNA
qRT‐PCR: quantitative real‐time polymerase chain reaction
RIP: RNA immunoprecipitation
TF: transcript factors
TV: tumor volume

## 1 Background

Colorectal cancer (CRC) is the third most common cancer (10.2% of all cancer cases) and second most common cause of cancer mortality (9.2% of all cancer mortality) globally [1]. In recent decades, the mortality rate of CRC has decreased due to the development of diagnostic techniques and treatment strategies, including surgery, adjuvant chemotherapies and palliative therapies. However, tumor metastasis remains the major obstacle in CRC treatment and prognosis. Liver metastasis is the most common site of distant spread and approximately 15–25% of CRC patients will have distant metastases at the time of primary diagnosis [2]. The importance of liver surgery specialist participation in multidisciplinary boards is now emphasized, but a 5‐year overall survival (OS) rate of 47–60% after hepatectomy for colorectal metastases is still not satisfactory [3, 4]. Moreover, recurrence occurs in 40–75% of patients after liver resection [5, 6]. Though previous research has identified a variety of molecules that play roles in the initiation and development of colorectal liver metastasis, the mechanisms remain unclear [7]. Therefore, understanding the underlying mechanisms of CRC and searching for highly sensitive and specific metastasis‐associated molecules, particularly during colorectal liver metastasis, are crucial to improve the clinical outcomes and OS of patients.

Circular RNA (circRNA) is a new type of non‐coding RNA (ncRNA) with a covalently closed loop and has no ability to code proteins. Compared with linear RNA, circRNA is more stable and is not affected by RNA exonucleases, deadenylation or cap removal [8, 9]. Emerging evidence has suggested that circRNA plays an essential role in complex human pathologies [10, 11]. Multiple studies also reveal that circRNA is involved in regulating the tumorigenesis and development of malignancies, including bladder cancer [12], gastric cancer [13], pancreatic cancer [14], hepatocellular carcinoma [15, 16] and non‐small cell lung cancer [17]. In 2019, we found that hsa_circ_101555 was significantly up‐regulated in CRC primary tumors in comparison with normal‐appearing tissues, which could be a useful prognostic indicator in CRC patients. It was concluded that circ101555 may function as a competing endogenous RNA (ceRNA) of miR‐597‐5p to up‐regulate CDK6 and RPA3 expressions [18]. Two recent review articles by Zeng and Dragomir systematically introduced the CRC‐related circRNA and their functional mechanisms, as well as the potential applications for CRC diagnosis and prognosis [19, 20]. Colorectal liver metastasis is a hotspot, and with the development of high‐throughput sequencing and novel computational technology, many novel circRNA have been verified to play a role in colorectal liver metastasis. Xu *et al*. [21] used the secondary sequencing to profile circRNA expression in the tissue samples from three CRC patients with liver metastasis and three matched CRC patients, and identified circRNA_0001178 and circRNA_0000826 as potential diagnostic biomarkers for colorectal liver metastasis. Chen *et al*. [22] also identified a novel and conserved circRNA (circ‐NSD2) as a promoter of CRC metastasis. Mechanistically, circ‐NSD2 acted as a sponge of the tumor suppressor miR‐199b‐5p, and activated *DDR1* and *JAG1* genes, which synergistically helped with cell‐‐matrix interaction, migration and metastasis of CRC cells. A similar study was also performed recently by Ren *et al*. [23]. They revealed the crucial role of hsa_circ_0001178 in CRC metastasis, and confirmed that CRC patients with high hsa_circ_0001178 levels were more prone to have metastatic clinical features, advanced TNM stages and adverse prognosis. A report from Chen *et al*. [24] revealed that N^6^‐methyladenosine modification of circNSUN2 modulated cytoplasmic export and stabilized HMGA2 to promote colorectal liver metastasis. However, these studies are limited, and the functions and mechanisms of circRNA in colorectal liver metastasis have not been fully elucidated.

In this study, the circRNA microarray was performed and hsa_circ_102049 (circ102049) was identified as a potential regulator of colorectal liver metastasis. The circ102049 expression in CRC samples and liver metastasis nodes was determined, and the prognostic role of circ102049 evaluated. Mechanistically, our subsequent experiments *in vitro* and *in vivo* demonstrated that circ102049 promoted the CRC progression through a miR‐761/miR‐192‐3p‐FRAS1‐dependent mechanism. Moreover, circ102049 may reduce mature miR‐761 and miR‐192‐3p expression indirectly due to the distribution of DGCR8 protein in cytoplasm. Our findings provide new clues that circ102049 may be a potential prognostic factor in CRC, and the circ102049‐miR‐761/miR‐192‐3p–FRAS1 axis could be further explored as an anti‐metastatic target for CRC patients.

## 2 Materials and methods

### 2.1 Tissues and cell lines

Paired samples of tumorous (Tumor), adjacent non‐tumorous tissues (Normal) and their corresponding metastatic liver nodes (Liver metastasis) were obtained from surgical resections of 202 CRC patients without preoperative treatment. Among them, 137 patients had no liver metastases and 65 were diagnosed as colorectal liver metastasis. All tissue specimens were collected from 2013 to 2015 and were immediately frozen in liquid nitrogen after surgical excision. The human material was obtained with the consent of patients and was approved by the ethics committee of the First Affiliated Hospital of Soochow University. Methodologies in the present study conform to the standards set by the Declaration of Helsinki.

Human CRC cell lines (SW1116, SW620, HCT116, DLD‐1, KM12, HT29 and LOVO) and human embryonic kidney cell 293T were purchased from American Type Culture Collection (ACTT, Manassas, VA, USA) and tested negative for mycoplasma contamination. Cells were cultured in RPMI‐1640 (Invitrogen, Carlsbad, CA, USA) or Dulbecco’s modified Eagle’s medium (Sigma, St. Louis, MO, USA) supplemented with 10% FBS (Gbico, Gaithersburg, MD, USA) at 37 °C in a humidified atmosphere with 5% CO_2_, and were in the logarithmic phase of growth for all experiments.

### 2.2 Microarray analysis: circRNA, miRNA and messenger (m)RNA microarray

Ten primary CRC tissues without liver metastasis and 10 primary CRC tumor tissues with liver metastasis were submitted to KangChen Bio‐tech (Shanghai, China) for circRNA and miRNA microarray, and to SHBIO Bio‐tech for mRNA microarray. The detail protocols were reported previously [18]. A fold‐change > 2 or < 0.5 was defined as statistically significantly different. All primary data in microarray analysis were uploaded to the NCBI Gene Expression Omnibus (https://www.ncbi.nlm.nih.gov/geo) with accession numbers GSE147597, GSE147602 and GSE147603.

### 2.3 Quantitative real‐time PCR

Total RNA was isolated from CRC tissues and cell lines using TRIzol reagent (Invitrogen). For circRNA and mRNA analysis, the PrimeScript RT Master Mix (Takara, Shiga, Japan) was used to synthesize cDNA. For miRNA analysis, specific cDNA was reverse‐transcribed using the RevertAid First Strand cDNA Synthesis (Thermo Scientific, Mountain View, CA, USA) and mature miRNA expression was assayed using TaqMan MicroRNA Assay (Applied Biosystems, Foster City, CA, USA) specific for hsa‐miR‐761 and miR‐192‐3p. PCR was performed using FastStart Universal SYBR Green Master (Roche, Mannheim, Germany) and analyzed by the LightCycler® 96 System (Roche). GAPDH was used as internal control for circRNA and mRNA detection, and U6 for miRNA evaluation. The relative expression levels were determined by the 2^−ΔCt^ or 2^−ΔΔCt^ method. All samples were processed in triplicate. Primers are listed in Table S1.

### 2.4 Fluorescence in situ hybridization

The fluorescence in situ hybridization (FISH) kit was purchased from RiboBio (Guangzhou, China) and the experiment was performed according to the manufacturer’s instructions [18]. The Cy3‐labeled circ102049 (5’‐CAGGAAAATCTGAAGTAGTGAAATGGAATGGCTGTG‐3’), FAM‐labeled miR‐761 (5’‐TGTGTCAGTTTCACCCTGCTGC‐3’) and FAM‐labeled miR‐192‐3p (5’‐CTGTGACCTATGGAATTGGCAG‐3’) probes were designed and synthesized by GenePharma (Shanghai, China). 18S and U6 probes were provided by RiboBio and images were obtained using a Zeiss (LSM510, Jena, Germany) confocal fluorescence microscope (LSM 510).

### 2.5 RNase R treatment

Total RNA 2 μg was incubated for 30 min at 37 °C with and without 5 U·μg^−1^ RNase R (Epicentre Technologies, Madison, WI, USA) and then purified by RNeasy MinElute Cleaning Kit (Qiagen, Germantown, MD, USA) and analyzed by quantitative real‐time PCR (qRT‐PCR).

### 2.6 Plasmids, small interfering (si)RNA and cell transfection

Human circ102049‐overexpressing vector (pLV‐circ102049‐Hygro) and the control plasmid were purchased from HarO Bio (Shanghai, China). A specially designed front circular frame and a back circular frame were obtained by PCR and then cloned into the pLV‐Hygro vector at the *Mlu*I and *Sal*l sites (named circ102049). Circ102049 was transiently silenced by siRNA specific to human circ102049 or FRAS1, designed and synthesized by GenePharma. The miR‐761, miR‐192‐3p mimics or inhibitors were also purchased from GenePharma (Table S2). The full‐length cDNA of DGCR8 was also amplified and cloned into the pcDNA3.1 vector. The above‐mentioned vectors were transfected by the Lipofectamine® 3000 transfection reagent (Invitrogen). After transfection for 48 h, the transfection efficiency of cells in each group was assessed by the qRT‐PCR analysis.

### 2.7 Bioinformatics analysis

The sequence of circ102049 was obtained from circbase (http://www.circbase.org). The miRNA which had potential binding sites of circ102409 were predicted by KangChen Bio‐tech. TargetScan (http://www.targetscan.org/) was used to predict the binding sites between miRNA and FRAS1. The cir102049/mRNA co‐expression analysis was based on calculating the Pearson correlation coefficient between the expression levels of mRNA and cir102049 in the human circRNA and mRNA array analysis. Difference integration analysis (Venn analysis) was used to show the common characteristic elements in the compared groups. Adhesion‐ or metastasis‐related mRNA, which might closely correlate with circ102049, were calculated by Venn analysis in circ102049 co‐expression mRNA, adhesion‐ or metastasis‐related genes in Genecards (http://www.genecards.org). Our microarray up‐regulated mRNA. Adhesion‐ or metastasis‐related genes are from PubMed. The common miRNA were obtained from 46 predicted circ102049‐binding miRNA (provided by KangChen Bio‐tech), 183 dysregulated miRNA by our miRNA microarray, and 966 FRAS1‐predicted miRNA (TargetScan). Gene set enrichment analysis (GSEA) was performed by the java program (http://software.broadinstitute.org/gsea/index.jsp) using MSigDB C2 CP: KEGG gene sets and gene symbols collection. GSEA, visualized using plug‐in the EnrichmentMap of cytoscape 3.6.0 software (http://cytoscape.org/), was used to determine whether the members of a given gene set were generally associated with sample type circ102049 expression (high versus low) or primary CRC tumors (with versus without liver metastasis). It was therefore conducted on all mRNA on the HG‐U133 Plus 2.0 ranked by enrichment score from most positive to most negative. A total of 1000 random sample permutations were carried out and the significance threshold set at FDR < 0.01.

### 2.8 CCK‐8, colony formation and apoptosis detection

Cell proliferation assay was performed using the cell counting kit‐8 (CCK‐8) reagent (Dojindo, Kumamoto, Japan). The transfected cells were seeded in 96‐well plates with 3 × 10^3^ cells per well and cultured for different time periods (0, 24, 48, 72 or 96 h). Each well was then supplemented with 20 μL CCK‐8 solution and incubated at 37 °C for 4 h. The optical density values at 450 nm were detected using the scan reader (ELx800; BioTek Instruments, Winooski, VT, USA).

For colony formation assay, 4 × 10^3^ cells were seeded in six‐well plates and incubated in complete culture medium containing 0.3% agar on top of 0.6% agar in the same medium at 37 °C. After 2 weeks, colonies were fixed with 70% ethanol and stained with 0.2% crystal violet. Colonies containing at least 50 cells were scored and observed under an inverted phase‐contrast fluorescence microscope IX71 (Olympus, Tokyo, Japan). Data are presented as the mean ± SD of five randomly scored fields.

Cell apoptotic rates were determined using the Annexin V Apoptosis Detection Kit PE according to the manufacturer’s instructions (Invitrogen). Briefly, cells were harvested, washed with 1× PBS and re‐suspended in 1× Binding Buffer to 1 × 10^6^ cells·mL^−1^. Annexin‐V‐PE 5 μL and PI 5 μL were added to 100 μL of the cell suspension and incubated for 15 min in the dark at room temperature. After incubation, 400 μL 1× Binding Buffer was added. Cells were analyzed using FACScan flow cytometer and each treatment was performed in triplicate.

### 2.9 Adhesion, migration and invasion assay

Briefly, treated cells (5 × 10^4^ each well) with Calcein AM (Keygene Biotech, Nanjing, China) were trypsinized, re‐plated on fibronectin‐coated coverslips (10 μg·mL^−1^) and incubated at 37 °C for 1 h. Following incubation, non‐adherent cells were removed by washing three times with PBS, and adherent cells were fixed with 200 μL PBS. The number of attached cells was determined by Calcein AM staining using a microplate reader and the adhesion activities were calculated as adherent cells/total cells × 100%.

Transwell chambers (8 μm, 24‐well insert; Corning, Lowell, MA, USA) were used for the migration assay. Briefly, 600 μL of medium containing 10% FBS was added to the lower chamber and 5 × 10^3^ cells in 200 μL of serum‐free medium to the upper chamber. Cells were incubated for 48 h at 37 °C. Non‐migrated cells were removed using cotton swabs. Finally, the insert membranes were stained with 1% crystal violet and the migrated cells were counted by microscope IX71 (Olympus). For the invasion assay, the insert membranes were coated with diluted Matrigel (BD Biosciences, San Diego, CA, USA). The other procedures were the same as for the migration assay.

### 2.10 Western blot

Total protein was extracted using RIPA Lysis Buffer (Beyotime, Shanghai, China) and protein concentration was detected by BCA Protein Assay Kit (Thermo Scientific). The protein extracts were then separated by 12% SDS/PAGE gel and transferred onto nitrocellulose membranes (Millipore, Billerica, MA, USA). After being blocked for 1 h, the membranes were incubated with primary antibodies at 4 °C overnight and horseradish peroxidase‐conjugated secondary antibodies (1 : 4000; Sigma) at room temperature for 2 h. The immunoreactive bands were detected with ECL kit (Thermo Scientific). Primary antibodies used are listed as follows: anti‐FRAS1 (1 : 1000, ab240583; Abcam, Cambridge, MA, USA), anti‐DGCR8 (1 : 1000, ab191875; Abcam). The band density was normalized to Histone H3 (1 : 1000, AH433; Beyotime) or GAPDH (1 : 2000, ab8245; Abcam) and quantified by imagej software.

### 2.11 RNA immunoprecipitation

The RNA immunoprecipitation (RIP) procedure was performed with a Magna RIP RNA‐Binding Protein Immunoprecipitation Kit (Millipore) according to the manufacturer’s instructions. CRC cells were lysed in RIP lysis buffer and then immunoprecipitated with antibodies with protein A/G magnetic beads. AGO2 (1 : 50, #2897, Cell Signaling Technology, Beverly, MA, USA), FRAS1 (1 : 100, ab240583; Abcam), DGCR8 (1 : 50, ab191875; Abcam) antibodies were used for RIP. Magnets were used to immobilize magnetic bead‐bound complexes while washing off the unbound materials. The immunoprecipitated RNA was extracted and measured by qRT‐PCR.

### 2.12 RNA pull‐down

RNA pull‐down assays were performed with Pierce™ Magnetic RNA‐Protein Pull‐Down Kit (Millipore). Biotin‐labeled control oligomers (UGCUUUGCACGGUAACGCCUGUUUU‐bio, known as Control probe) or an oligomer complementary to the sequence of circ102049 (CAGGAAAAUCUGAAGUAGUGAAAUGGAAUGGCUGUG‐bio, also known as circ102049 probe) from GenePharma, Shanghai, China, were synthesized and utilized, incubated with the lysates from SW1116 cells for 2 h at 25 °C. The RNA/protein or RNA/miRNA complexes were then captured with streptavidin‐coupled dynabeads for 1 h at 25 °C. The complexes in the pull‐down were determined using Western blot or qRT‐PCR analysis.

### 2.13 Chromatin isolation by RNA purification

Chromatin isolation by RNA purification (ChIRP) assay is the suitable approach to find a target DNA that is directly regulated by circ102049. A Magna ChIRP RNA Interactome Kit (Millipore) was used to perform a ChIRP experiment according to the manufacturer’s protocol. The CHIRPed DNA was purified using the QIAquick PCR Purification Kit (Qiagen). The primer sequences targeting FRAS1 promoter are listed in Table S3. The FRAS1 promoters in the pull‐down were then determined using qRT‐PCR analysis.

### 2.14 Luciferase

The wild‐type predictive binding sites or mutant binding sites of circ102049 with miR‐761 or miR‐192‐3p were cloned into the luciferase reporter psiCHECK2 (Promega, Madison, WI, USA) vector. The plasmids were then co‐transfected with miR‐761 (or miR‐192‐3p) mimics into SW620 cells. FRAS1‐3’UTR with a wild‐type or mutant seed region were cloned into pMIR‐REPORTER vector (Thermo Scientific) and cotransfected with miR‐761 and miR‐192‐3p mimics, respectively, to confirm the direct binding between miR‐761 (or miR‐192‐3p) and FRAS1. At 48 h after transfection, the luciferase activity was detected and firefly luciferase activity was normalized relative to Renilla luciferase activity.

### 2.15 Animal studies

Four‐week‐old male BALB/c nude mice were purchased from Shanghai SLAC Laboratory Animal Company (Shanghai, China) and divided into four groups (five mice per group) as follows: NC, circ102049, small interfering (si)‐FRAS1 and circ102049 + si‐FRAS1 group. Aliquots of 200 μL of 4 × 10^6^ SW1116 cells in infected with lentiviral NC, lentiviral‐circ102049, lentiviral‐short hairpin (sh)‐FRAS1 or with lentiviral‐circ102049 and lentiviral‐sh‐FRAS1 were injected subcutaneously into the flanks of nude mice. Tumor volume (TV) was measured weekly and calculated as follows: TV (mm^3^) = length × width^2^ × 0.5. Mice were sacrificed 4 weeks after inoculation and the tumor tissues were excised and stored at −80 °C for further qRT‐PCR analysis. To observe the metastasis *in vivo*, four groups were again formed and mice were injected into the tail with 200 μL of 4 × 10^6^ SW1116 cells. After 1.5 months, mice were sacrificed and the lungs and livers collected and photographed. Lung and liver metastatic nodes per mouse were detected and calculated. Animal studies were approved by the Institutional Animal Care and Use Committee of the First Affiliated Hospital of Soochow University.

### 2.16 Statistical analysis

The measurement data are presented as mean ± standard deviation, and all statistical analyses were performed using spss 20.0 (IBM, SPSS, Chicago, IL, USA). Comparisons between groups were undertaken using Student’s *t*‐test or one‐way ANOVA. OS curves were calculated using the Kaplan–Meier method and analyzed with the log‐rank test. The univariate and multivariate analyses were analyzed by Cox proportional hazards model. The correlation between two variables was determined using the Pearson correlation test. *P* < 0.05 was considered statistically significant.

## 3 Results

### 3.1 Identification of circ102049 in CRC primary tissues via microarray analysis

**Fig 1:**
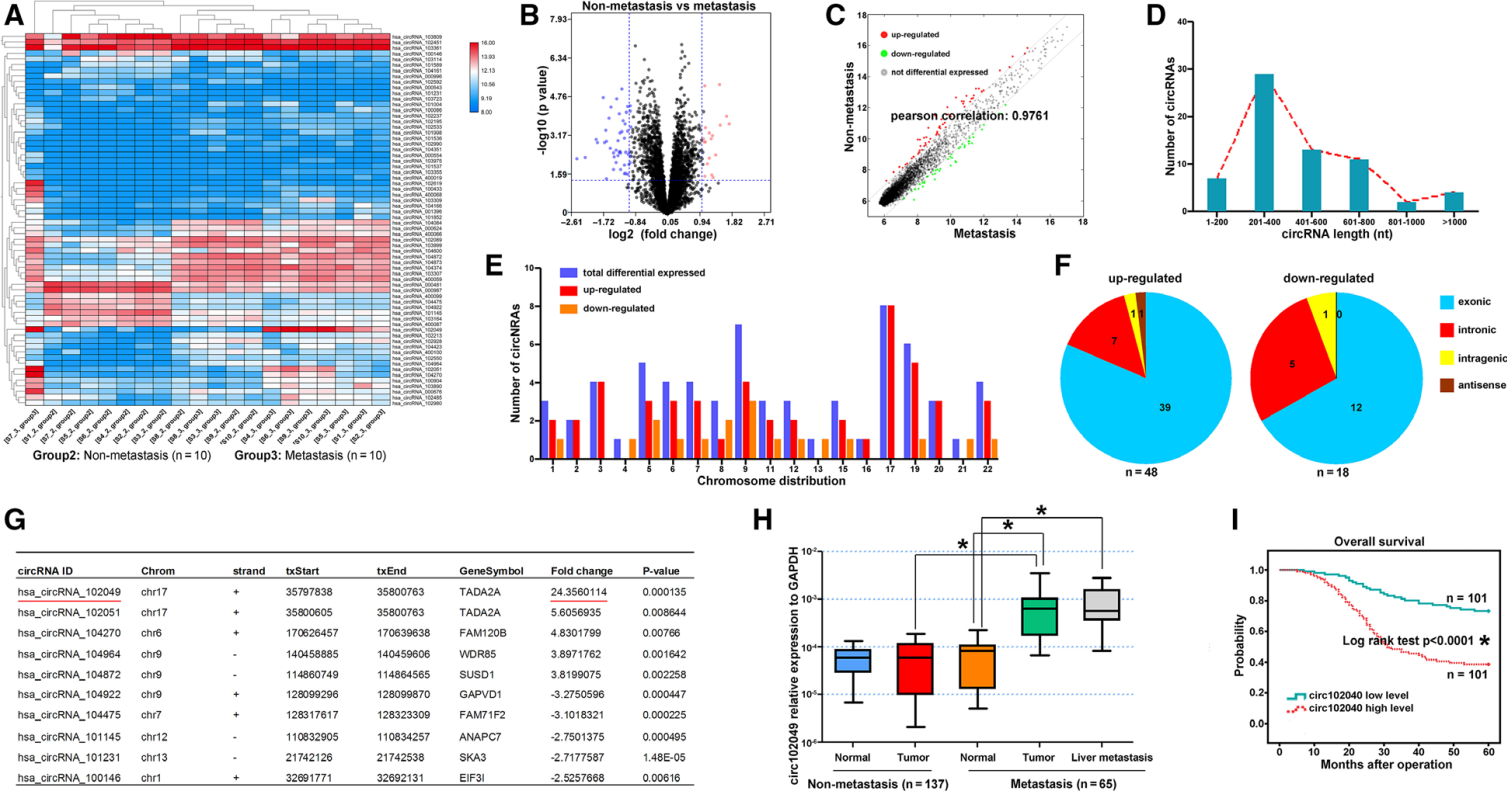
Identification of circ102049 in CRC primary tissues via microarray analysis. (A–C) CircRNA expression profiles between 10 primary CRC tumors without liver metastasis and 10 primary tumor tissues with liver metastasis through circRNA microarray were performed, and 66 dysregulated circRNA were identified by hierarchical clustering, volcano and scatter plot. (D–F) The length distribution, chromosome map of the human genome and genomic origin of these 66 circRNA. (H) Circ102049 expression in 202 CRC patients with (*n* = 65) and without liver metastasis (*n* = 137) were determined by qRT‐PCR. (G) OS curves were plotted, and the data showed that CRC patients with high circ102049 levels had a worse 5‐year OS, compared with those patients with low expressions (*P < 0.05; error bars represent standard deviation).

To characterize liver metastasis‐related circRNA expression profiles in CRC tissues, we compared 10 primary CRC tissues without liver metastasis and 10 primary CRC tissues with liver metastasis using circRNA microarray. A total of 66 circRNA were dysregulated in primary tumor tissues when liver metastases occurred, of which 48 circRNA were up‐regulated and 18 down‐regulated. These 66 differentially expressed circRNA are shown by hierarchical clustering, volcano and scatter plot, respectively (Fig. 1A–C). By bioinformatics analysis, the length of most circRNA was 201–400 nucleotides, and these 66 circRNA were widely distributed on diverse chromosomes and mostly located at exonic regions (Fig. 1D–F). The top five up‐ or down‐regulated circRNA were listed, and circ102049 was considered the most up‐regulated circRNA (24.36‐fold change, Fig. 1G). Next, we examined circ102049 expression in 202 CRC patients with (*n* = 65) and without liver metastasis (*n* = 137) using qRT‐PCR. Our results showed that no changes of circ102049 were found between CRC primary tumors without liver metastasis and the matched controls, but its expression in CRC primary tumors with liver metastasis and in their corresponding liver metastatic nodes was significantly up‐regulated compared with the matched controls (Fig. 1H). In addition, OS curves were plotted; the data showed that CRC patients with high circ102049 levels had a worse 5‐year OS compared with those patients with low circ102049 levels (*P < 0.001, Fig. 1I). These results strongly suggest that circ102049 might serve as a metastasis‐related ncRNA and that it is closely correlated with the prognosis of CRC patients.

### 3.2 Characteristics of circ102049 in CRC cells

**Fig 2:**
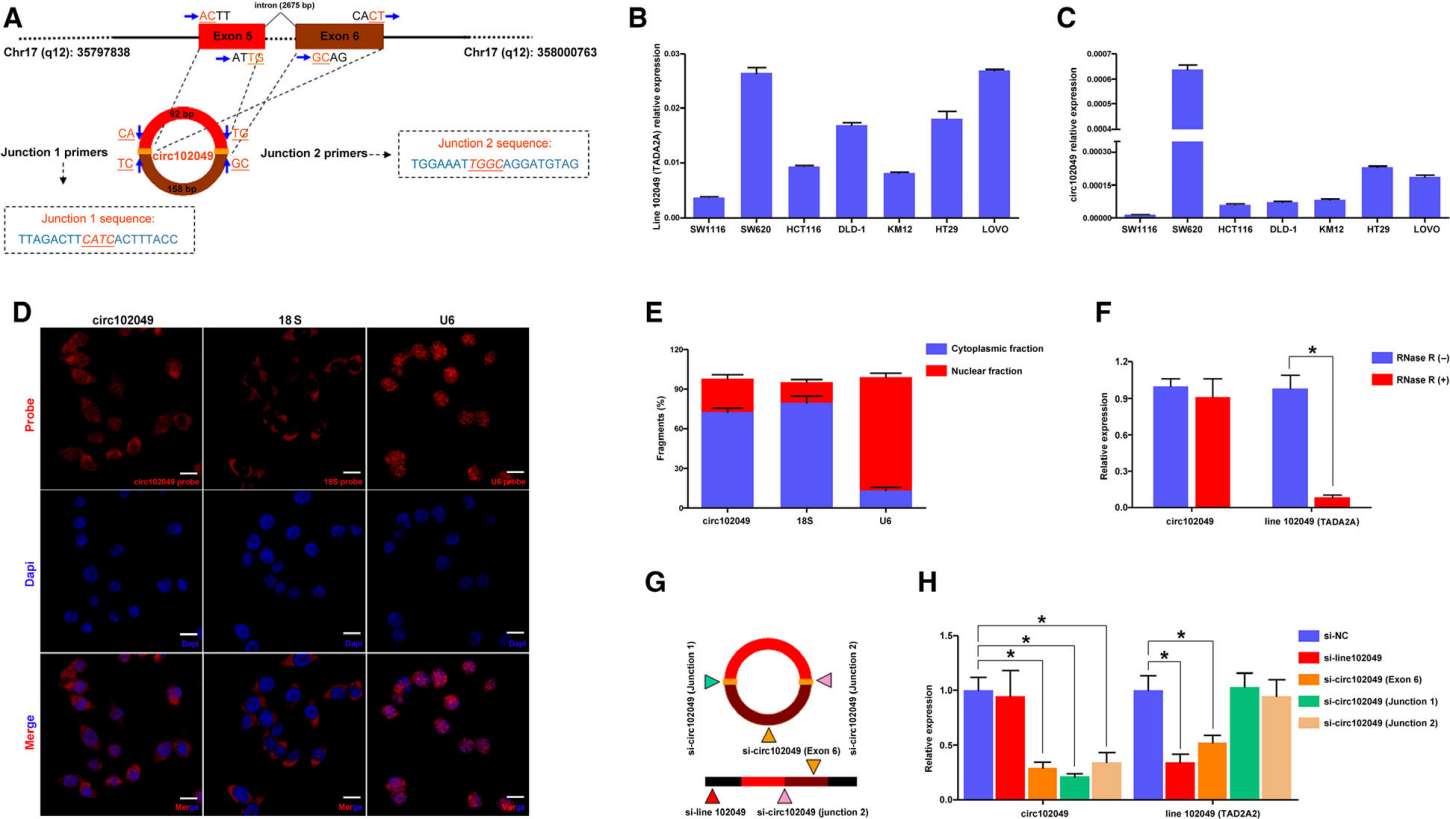
Characteristics of circ102049 in CRC cells. (A) Circ102049 arose from the *TADA2A* gene (line 102049) and consisted of two exons. The back‐spliced (junction 1) and joint part (junction 2) of circ102049 were confirmed by the Sanger sequencing. (B,C) The relative common expressions between circ102049 and linear 102049 in different CRC cells were detected by qRT‐PCR. (D,E) The location of circ102049, 18S and U6 in SW620 cells were observed by the FISH assay; Scale bar = 10 μm. (F) SW620 cells were treated with RNase R and circ102049 stability was analyzed. (G,H) Interference fragments which specifically targeted the linear 102049, exon 6, junction 1 and 2 of circ102049 were synthesized and transfected into SW620 cells. Expressions of circ102049 and linear 102049 were determined by qRT‐PCR (*P < 0.05; error bars represent standard deviation).

Circ102049 is also known as hsa_circ_0043278 (https://circinteractome.nia.nih.gov/) and arises from the *TADA2A* gene (Gene ID: 6871, https://www.ncbi.nlm.nih.gov/gene/?term=6871, chr17: 35766977–35839830). The genomic length of line 102049 (chr17: 35797838–35800763) is 2925 bp, and circ102049 consists of the head‐to‐tail splicing of exon 5 and 6 with a length of 250 bp. In our study, the back‐spliced (junction 1) and joint part (junction 2) of circ102049 were specifically amplified by divergent and convergent primers, respectively, in SW620 cells, and Sanger sequencing of the PCR products confirmed the presence of splice junctions in circ102049 (Fig. 2A). The results of qRT‐PCR also showed that there were relatively common expressions between circ102049 and linear 102049 in different CRC cells (Fig. 2B,C). From the general information of seven CRC cells included in our study (Table S4), SW620 and LOVO cells, derived from the metastatic sites of CRC patients, showed relatively high levels of line 102049 and circ102049, in comparison with other non‐metastasis‐derived cell lines. To uncover further the location of circ102049 in CRC cells, we designed and synthesized the specific probes of circ102049. The confocal microscopic observation of FISH assay revealed that circ102049 was primarily expressed in the cytoplasm of SW620 cells, similarly to 18S as controls (Fig. 2D,E). Next, circ102049 stability was analyzed and SW620 cells were treated with RNase R. qRT‐PCR indicated that circ102049 resisted digestion after the treatment of RNase R exonuclease in comparison with the linear RNA, further implying that circ102049 possesses a stable loop structure (Fig. 2F). In addition, interference fragments which specifically targeted the linear 102049, exon 6, junctions 1 and 2 of circ102049 were synthesized and transfected into SW620 cells. The results demonstrated that linear 102049 could be attenuated by si‐line 102049 and circ102049 (Exon 6). In contrast, circ102049 was significantly knocked down by targeting Exon 6, junction 1 or 2 (Fig. 2G,H). The above results suggested that circ102049 was derived from the host gene *TADA2A* and had a stable loop structure based on head‐to‐tail splicing.

### 3.3 Circ102049 promotes the adhesion, migration and invasion of CRC cells

**Fig 3:**
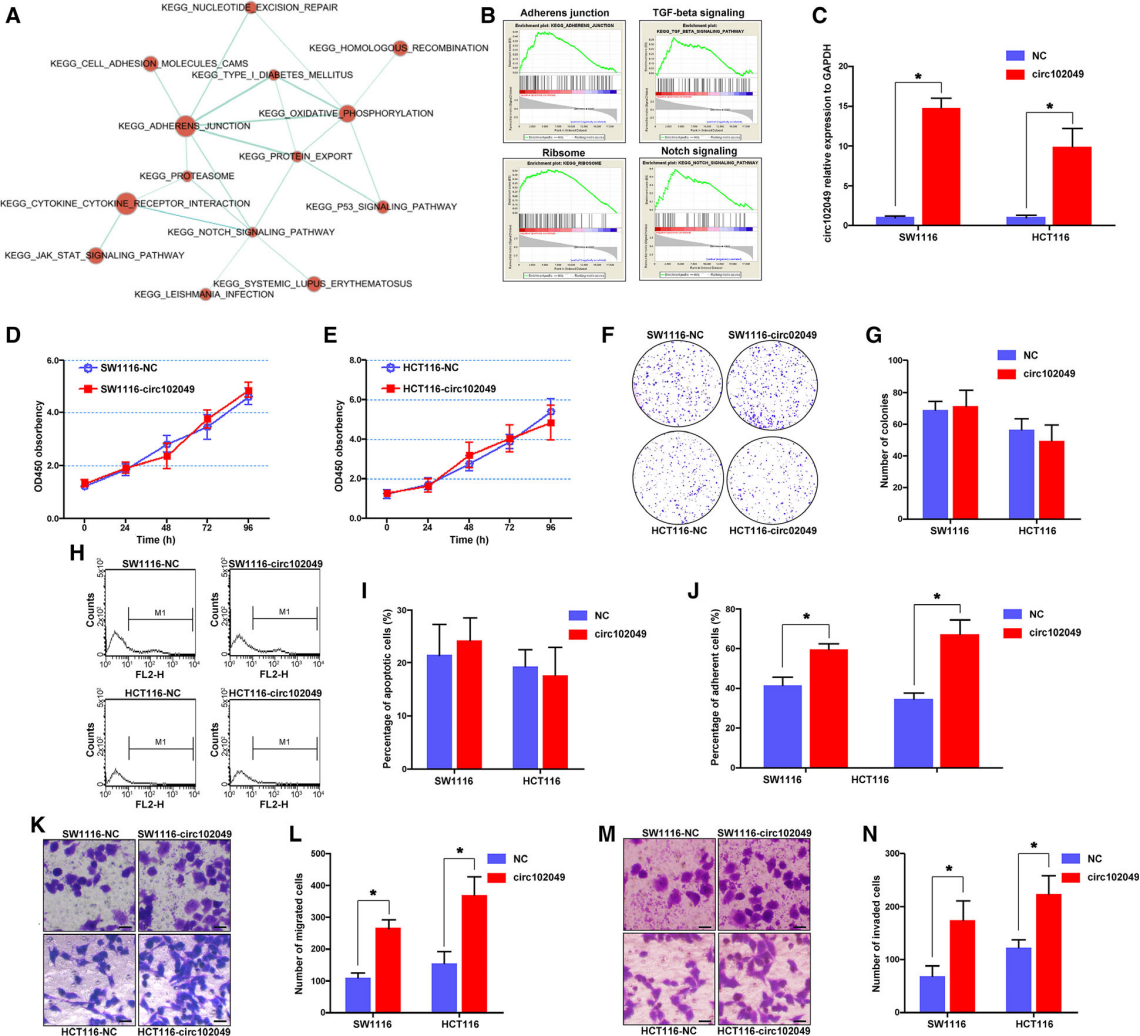
Circ102049 promoted the adhesion, migration and invasion of CRC cells. (A,B) GSEA analysis showed that metastasis‐related signaling such as adherens junctions, TGF‐β signaling, ribosome and Notch signaling were closely correlated with circ10249. (C) The circ102049 overexpression vector was efficient by qRT‐PCR analysis. (D,E) The proliferative ability was determined by the CCK‐8 assay. (F,G) The colony formation assay was performed and calculated. (H,I) The apoptotic rates of CRC cells were determined through Annexin‐V staining. (J) The ability of cell adhesion was determined. (K–N) The ability of cell migration and invasion was determined; Scale bar = 10 μm (*P < 0.05; error bars represent standard deviation).

To determine the biological functions and pathways affected by circ102049 in CRC, we subjected microarray data to GSEA analysis. The results showed that metastasis‐related signaling such as adherens junctions, TGF‐β signaling, ribosome and Notch signaling were closely correlated with circ10249 (Fig. 3A,B). The circ102049 and mock expression vectors were constructed and the PCR results indicated that the circ102049 overexpression vector was efficient for our subsequent studies (Fig. 3C). Through CCK‐8 assay and colony formation assay, in comparison with the control groups, the proliferation abilities of SW1116 and HCT116 cells were not affected by circ102049 (Fig. 3D–G). Annexin V staining was also performed further to confirm that circ102049 could not influence the apoptotic rates of CRC cells (Fig. 3H,I). We performed the cell adhesion, migration and invasion assays to assess the effects of circ201049 on tumor metastasis. As shown in Fig. 3J–N, overexpression of circ102049 could significantly increase the adhesion, migrating and invasive capacities of CRC cells. Simultaneously, circ102049 was effectively silenced by siRNA (si‐circ102049, Junction 1) in SW620 and HT29 cells (Fig. S1A). The biological functions of circ102049 on CRC cells were also examined. Similarly, no changes of proliferation and apoptosis were observed when circ102049 was knocked down in CRC cells (Fig. S1B–G). However, silencing circ102049 significantly suppressed the cell adhesion, migration and invasion in both SW620 and HT29 cells (Fig. S1H–L). These results indicated that circ102049 may play a metastasis‐promoting role in CRC.

### 3.4 Circ102049 promotes the tumor metastasis through a FRAS1‐dependent mechanism

**Fig 4:**
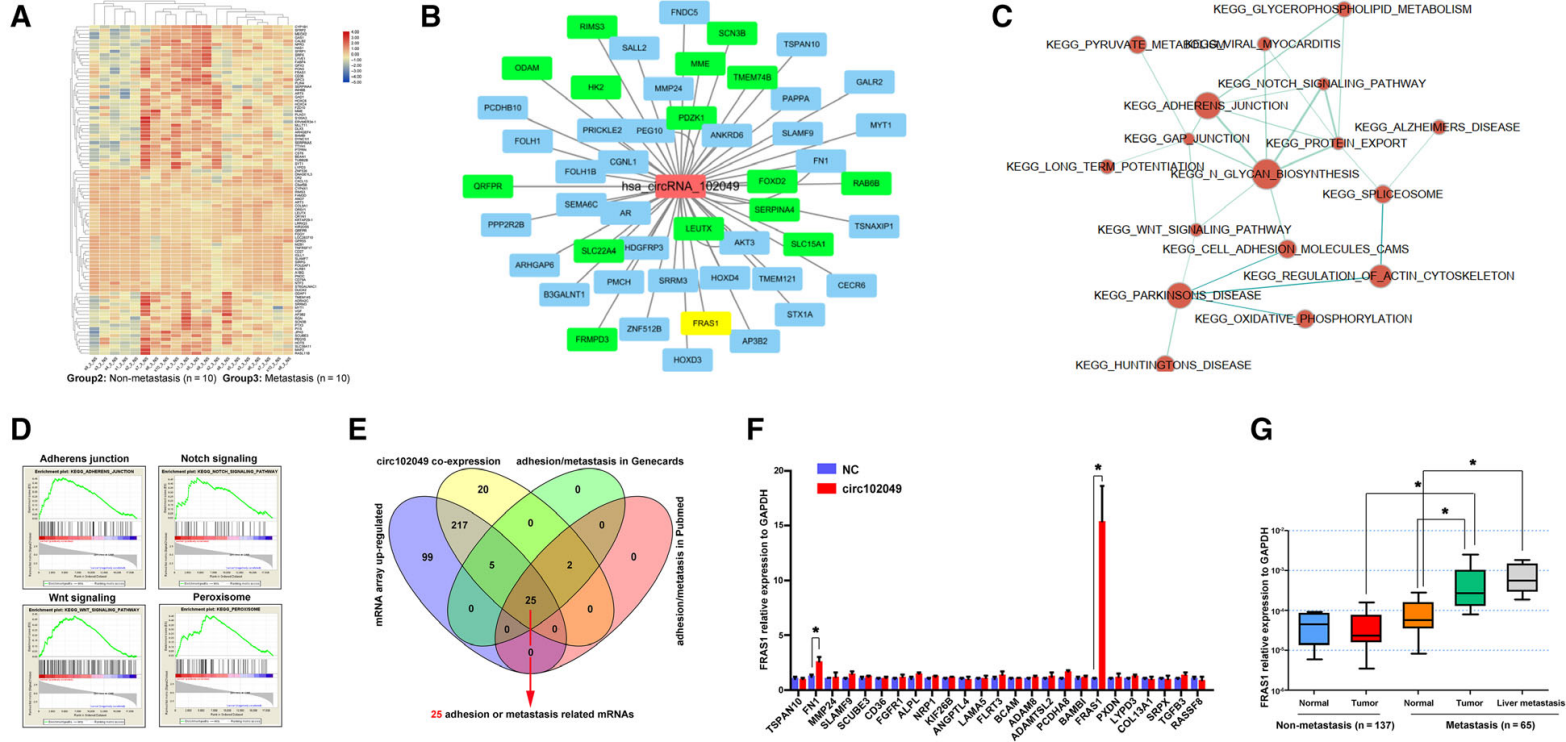
Circ102049 promoted the tumor metastasis through a FRAS1‐dependent mechanism. (A) mRNA expression profiles between 10 primary CRC tumors without liver metastasis and 10 primary tumor tissues with liver metastasis through mRNA microarray were performed, and 484 dysregulated mRNA were identified by hierarchical clustering. (B) The co‐expression analysis presented the potential interacted genes of circ102049. (C,D) GSEA analysis showed that metastasis‐related signaling such as adherens junction, Notch signaling, Wnt signaling and peroxisome were involved in colorectal liver metastasis. (E) Venn analysis showed the common characteristic elements among the compared groups; 25 genes might be correlated with circ102049. (F) qRT‐PCR analysis confirmed that FRAS1 was the most significantly up‐regulated gene after circ102049 overexpression in SW1116 cells. (G) The FRAS1 mRNA expression in 202 CRC patients with (*n* = 65) and without liver metastasis (*n* = 137) was determined by qRT‐PCR (*P < 0.05; error bars represent standard deviation).

To investigate further the underlying mechanism and search the potential downstream molecules of circ102049, a corresponding mRNA microarray was performed between primary CRC tumors with and those without liver metastasis. A total of 484 dysregulated mRNA were involved in the liver metastasis, and co‐expression analysis presented the potential interacting genes of circ102049 (Fig. 4A,B). Meanwhile, the GSEA analysis between non‐metastasis and metastasis groups was conducted. The results showed that these metastasis‐related functions or signals, such as adherens junction, Notch signaling, Wnt signaling and peroxisome, were involved in colorectal liver metastasis (Fig. 4C,D). Difference integration analysis (Venn analysis) was used to show the common characteristic elements among the compared groups; 25 genes, including *TSPAN10*, *FN1*, *MMP24* and *FRAS1*, may be correlated with circ102049 (Fig. 4E). The results of our qRT‐PCR analysis confirmed that only two molecules (FN1 and FRAS1) may be involved; *FRAS1* was the most significantly up‐regulated gene after circ102049 overexpression in SW1116 cells (Fig. 4F). To reveal the potential role of FRAS1 in CRC, we determined its mRNA expression in 202 CRC tissues. Our data demonstrated that the FRAS1 levels were significantly elevated in primary tumors and liver metastatic nodes in CRC patients with liver metastasis, whereas no expression changes were observed between CRC primary tumors without liver metastasis and the matched controls (Fig. 4G). These data imply that FRAS1 may be the key downstream target of circ1020049 and promotes liver metastasis in clinical patients.

To investigate further whether circ102049 was involved in CRC progression by regulating FRAS1, a series of rescue assays were employed. We first reduced the expression of FRAS1 by siRNA fragments (Fig. S2A). The proliferation of CRC cells was examined. As expected, si‐FRAS1 treatment did not change the proliferation of SW620 and HT29 cells, and the proliferation of CRC cells transfected with circ102049 + si‐FRAS1 was also not influenced in comparison with those transfected with circ102049 (Fig. S2B–E). Similar apoptotic rates were also observed in SW620 and HT29 cells (Fig. S2F–G). Lastly, the metastatic abilities of CRC cells were evaluated. The results showed that si‐FRAS1 could significantly suppress adhesion, migration and invasion in SW620 and HT29 cells. More importantly, si‐FRAS1 treatments could counteract the circ102049 overexpression‐mediated promotion of cell adhesion, migration and invasion (Fig. S2H–J). In a word, circ102049 could promote adhesion, migration and invasion of CRC cells via a FRAS1‐dependent mechanism.

### 3.5 Circ102049 up‐regulated FRAS1 expression by sponging miR‐761 and miR‐192‐3p

To better reveal the relationships between circ102049 and FRAS1, we first silenced circ102049 expressions by si‐circ102049 (Junction 1) fragments. PCR and Western blot analysis (Fig. S3A,B) showed that circ102049 could significantly regulate FRAS1 in both mRNA and protein levels, indicating that circ102049 may interact directly with FRAS1 or might regulate FRAS1 indirectly through other molecules such as transcript factors (TF). Unfortunately, the results of RNA pull‐down and RIP assay all implied that there were no direct interactions between circ102049 and FRAS1 (Fig. S3C,D). Using a circ102049‐specific probe, we also found by CHIRP assay that circ102049 could not enrich the FRAS1 promoter region (−2000 to 0 bp) from the transcription start site (Fig. S3E).

**Fig 5:**
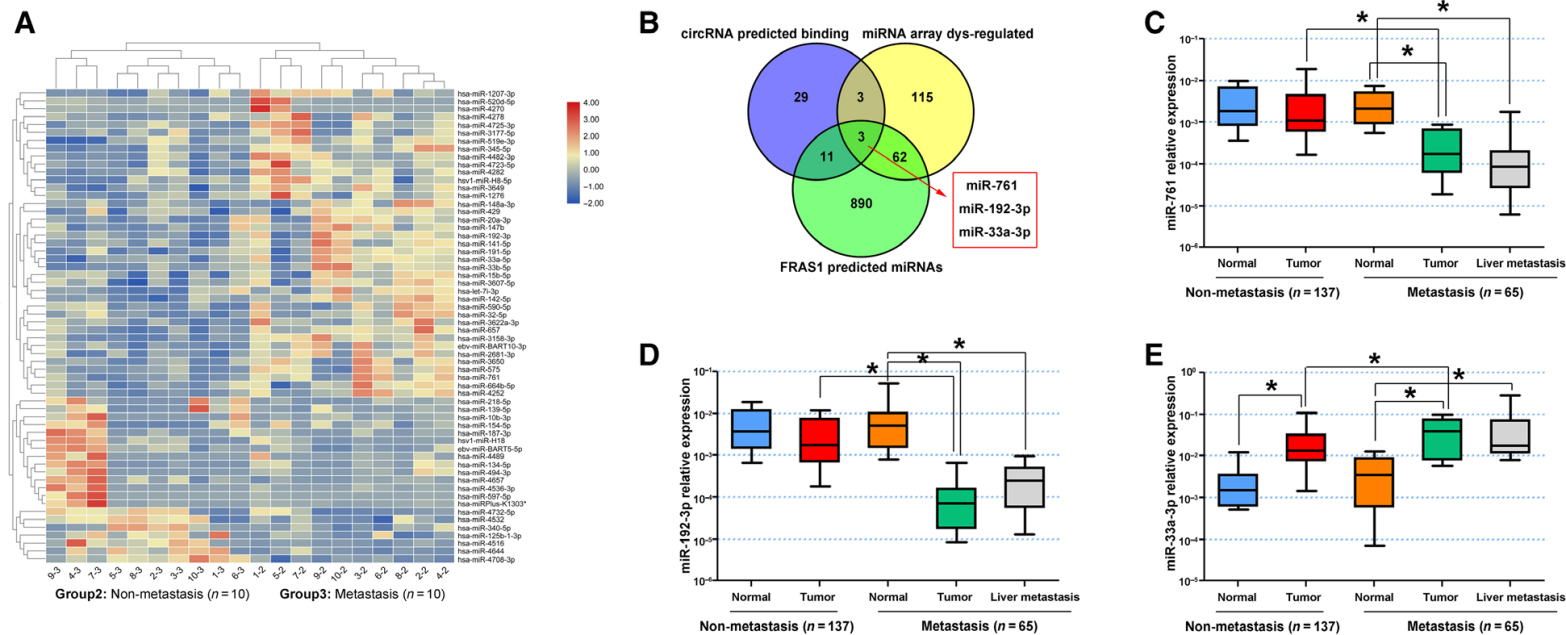
Circ102049 up‐regulated FRAS1 expression by sponging miR‐761 and miR‐192‐3p. (A) A miRNA array was performed to analyze the differentially expressed miRNA between primary CRC tumors with and without liver metastasis; 183 miRNA were identified by hierarchical clustering. (B) Venn analysis showed the common characteristic miRNA among the compared groups; miR‐761, miR‐192‐3p and miR‐33a‐3p might participate in the ceRNA regulation of circ102049. (C–E) miR‐761, miR‐192‐3p and miR‐33a‐3p expressions in 202 CRC patients with (*n* = 65) and without liver metastasis (*n* = 137) were determined by qRT‐PCR (*P < 0.05; error bars represent standard deviation).

Based on the nuclear‐cytoplasmic fractionation by FISH assay, circ102049 was mainly discovered in the cytoplasm. Hence, we hypothesized that circ102049 may function as a ceRNA in CRC. To support our hypothesis, a miRNA microarray was performed to analyze the differentially expressed miRNA between primary CRC tumors with and without liver metastasis, identifying a total of 183 miRNA (Fig. 5A). Combined with the circ102049‐predicted binding of miRNA (n = 46) and potential FRAS1‐regulated miRNA by TargetScan (*n* = 966), Venn analysis concluded that only three miRNA (miR‐761, miR‐192‐3p and miR‐33a‐3p) may participate in the competing endogenous (ce)RNA regulation of circ102049 (Fig. 5B). Of these three miRNA, only miR‐761 and miR‐192‐3p levels in CRC primary tumors or liver metastatic nodes were significantly down‐regulated in comparison with the normal tissues or non‐metastatic primary CRC tumors (Fig. 5C–E).

To confirm our hypothesis of ceRNA, we predicted the potential binding sites between circ102049 and miR‐761 (or miR‐192‐3p; Fig. S4A). There was a surprising reduction of luciferase activity in co‐transfected cells with the miR‐761 (or miR‐192‐3p) mimics and circ102049‐wild‐type (p‐luc‐circ102049) reporter genes (Fig. S4B,C). The FISH results showed that circ102049 and miR‐761 (or miR‐192‐3p) were preferentially co‐localized in the cytoplasm of SW620 cells (Fig. S4D). The anti‐Ago2 RIP results showed that endogenous circ102049 pull‐down by AGO2 was specifically enriched in SW620 cells upon overexpression of miR‐761 or miR‐192‐3p (Fig. S4E,F). Meanwhile, the RNA pull‐down analysis demonstrated that endogenous miR‐761 or miR‐192‐3p could also be significantly pulled down by biotinylated probes against circ102049 (Fig. S4G–I). These data strongly validated the direct binding of circ102049 with miR‐761 and miR‐192‐3p in CRC cells.

The downstream regulatory relationships of miR‐761 (miR‐192‐3p) and FRAS1 were also discussed. First, the binding sites between miR‐761 (or miR‐192‐3p) and FRAS1 were predicted by TargetScan (Fig. S5A). The Western blot analysis showed that transfection of miR‐761 or miR‐192‐3p precursor led to a significant decrease of FRAS1 protein in SW620 cells (Fig. S5B,C). In addition, the luciferase reporter assay was used to determine whether these two miRNA could target FRAS1 3’‐UTR sites. The data revealed that a considerable reduction of luciferase activity was observed when it was co‐transfected with miR‐761 mimics and FRAS1‐wild type (or mutant‐1 type: mutant sites form the position of 452–458 of FRAS1 3’‐UTR). Similar results were also observed when miR‐192‐3p mimics and FRAS1‐wild type (or mutant‐2 type: mutant sites form the position of 3346–3352 of FRAS1 3’‐UTR). This data demonstrated that miR‐761 and miR‐192‐3p could specifically target FRAS1 3’‐UTR on the sites of 2271–2277 and 1879–1885, respectively (Fig. S5D,E). In short, *FRAS1* was confirmed as a common target gene of miR‐761 and miR‐192‐3p in CRC cells.

Considering the results *in vitro* that circ102049 may up‐regulate FRAS1 expression through sponging miR‐761 and miR‐192‐3p, we further analyzed the expression relationships of these four molecules in 202 CRC tissues. As presented in Fig. S6A–E, the circ102049 levels in tumor tissues were positively associated with the FRAS1 mRNA expressions but negatively associated with the mature miR‐761 or miR‐192‐3p levels. Similarly, the levels of these two miRNA were negatively associated with their target gene FRAS1. Univariate analysis for OS showed that lymph node metastasis, circ102049 expression and FRAS1 were all prognostic factors for poor prognosis. Variables with a *P*‐value < 0.05 by univariate analysis were selected for multivariate analysis using Cox’s proportional hazards model. Multivariate analysis showed that only high expressions of circ102049 [hazard ratio (HR) 2.021, 95% CI 1.292–3.161; *P* = 0.002] and FRAS1 (HR 1.87, 95% CI 1.166–3; *P* = 0.009) were retained as independent and significant prognostic factors for survival (Table S5). All such data from clinical tissues are further evidence that circ102049 sponged miR‐761 and miR‐192‐3p to regulate the FRAS1 mRNA expressions in CRC.

### 3.6 Circ102049 distributes DGCR8 to down‐regulate the mature miR‐761 and miR‐192‐3p expressions

**Fig 6:**
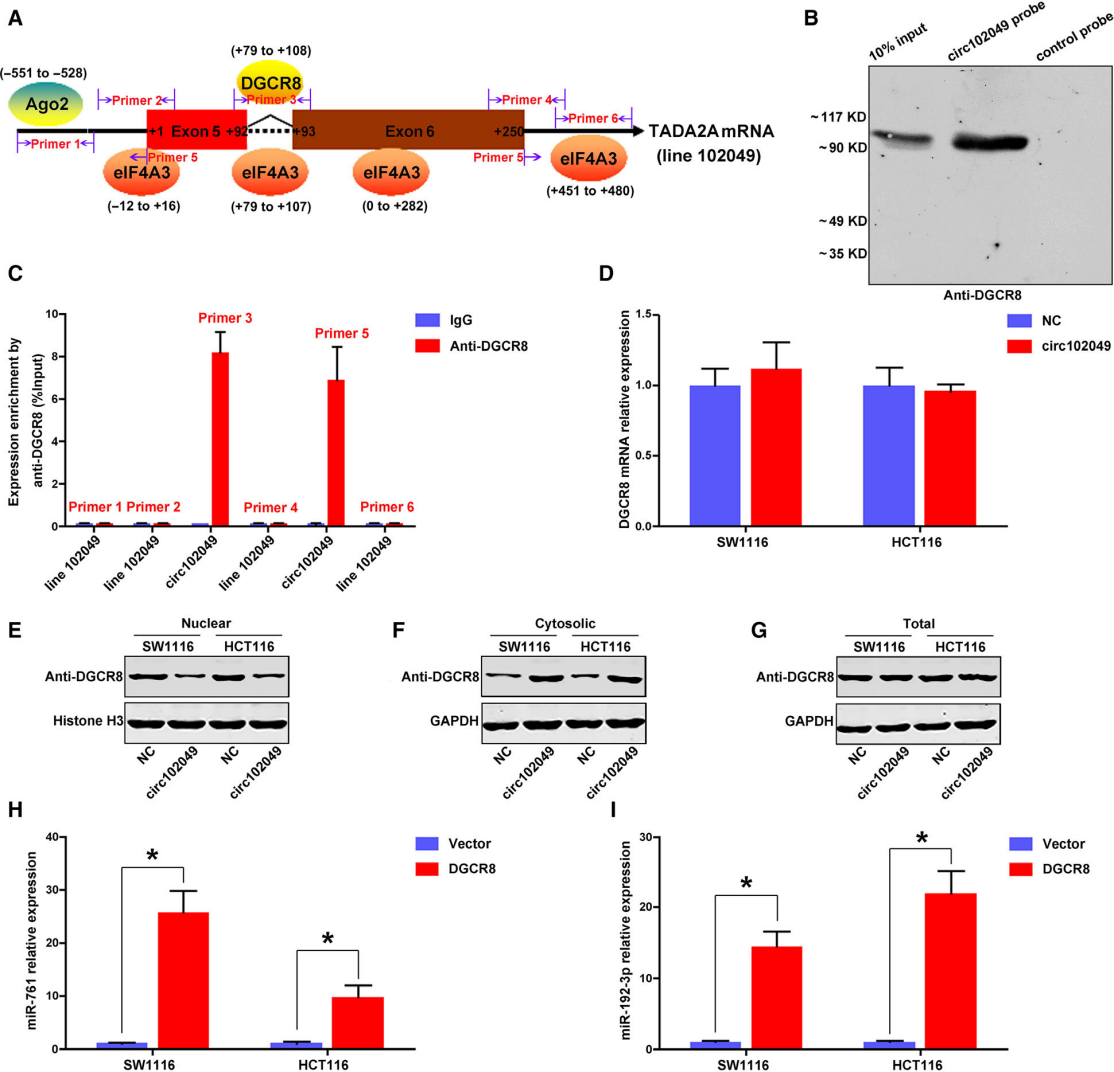
Circ102049 distributed DGCR8 to down‐regulate the mature miR‐761 and miR‐192‐3p expression in CRC cells. (A) Binding site of DGCR8 on the circ102049 mRNA transcript was predicted by circRNA interactome. (B) RNA pull‐down assay using a specific circ102049 probe could effectively enrich DGCR8 protein in SW620 cells, compared with the control probe. (C) RIP assay using anti‐DGCR8 antibody indicated that DGCR8 could directly and specifically bind to circ102049. (D) DGCR8 mRNA expressions after circ102049 overexpression in CRC cells were determined by qRT‐PCR. (E–G) Nuclear, cytosolic and total DGCR8 proteins after circ102049 overexpression in CRC cells were determined by Western blot. (H,I) Mature miR‐761 and miR‐192‐3p expressions in CRC cells after DGCR8 overexpression were determined by qRT‐PCR (*P < 0.05; error bars represent standard deviation).

Using the bioinformatics method (https://circinteractome.nia.nih.gov/index.html), we found that one binding site for DGCR8 was present in the downstream region of the circ102049 mRNA transcript (Fig. 6A). The RNA pull‐down assay using a specific circ102049 probe could effectively enrich DGCR8 protein in SW620 cells compared with the control probe (Fig. 6B). We then designed and synthesized six pairs of primers which could specifically amplify the linear 102049 (Primers 1, 2, 4 and 6) and circ102049 (Primers 3 and 5) products. The RIP assay using anti‐DGCR8 antibody indicated that DGCR8 could directly bind to circ102049 (Fig. 6C). To reveal further the relationships between circ102049 and DGCR8 protein in CRC cells, we examined the DGCR8 mRNA expressions after circ102049 overexpression in both SW1116 and HCT116 cells. The results of PCR analysis showed that no changes were found (Fig. 6D). However, the number of nuclear DGCR8 proteins were decreased and the cytosolic levels were elevated after circ102049 overexpression in CRC cells. More interestingly, total protein expressions of DGCR8 were not influenced (Fig. 6E–G). The co‐transcriptional cleavage of primary miRNA (pri‐miRNA) to precursor miRNA (pre‐miRNA) is controlled by a variety of mechanisms, including signal‐induced Drosha interaction with pri‐miRNA chromatin, RNA‐binding protein‐assisted recruitment of DGCR8/Drosha to the pri‐miRNA, and regulation of Drosha/DGCR8 expression. Therefore, DGCR8 protein is essential for the biogenesis of miRNA. In this study, we also determined the DGCR8 mRNA expression in 202 CRC patients. The results implied that there were no changes of DGCR8 mRNA expressions between CRC primary tumors (or liver metastatic nodes) and paired normal tissues (Fig. S7). But our results of PCR analysis confirmed that DGCR8 overexpression could significantly influence mature miR‐761 and miR‐192‐3p expression in CRC cells (Fig. 6H,I). Considering the locations of circ102049 and DGCR8 protein in previous studies, we speculated that mature circ102049 in cytoplasm could effectively sponge DGCR8 proteins out of the nucleus, and ultimately reduce the mature products of miR‐761 and miR‐192‐3p.

### 3.7 Circ102049 promotes the colorectal liver metastasis *in vivo*

**Fig 7:**
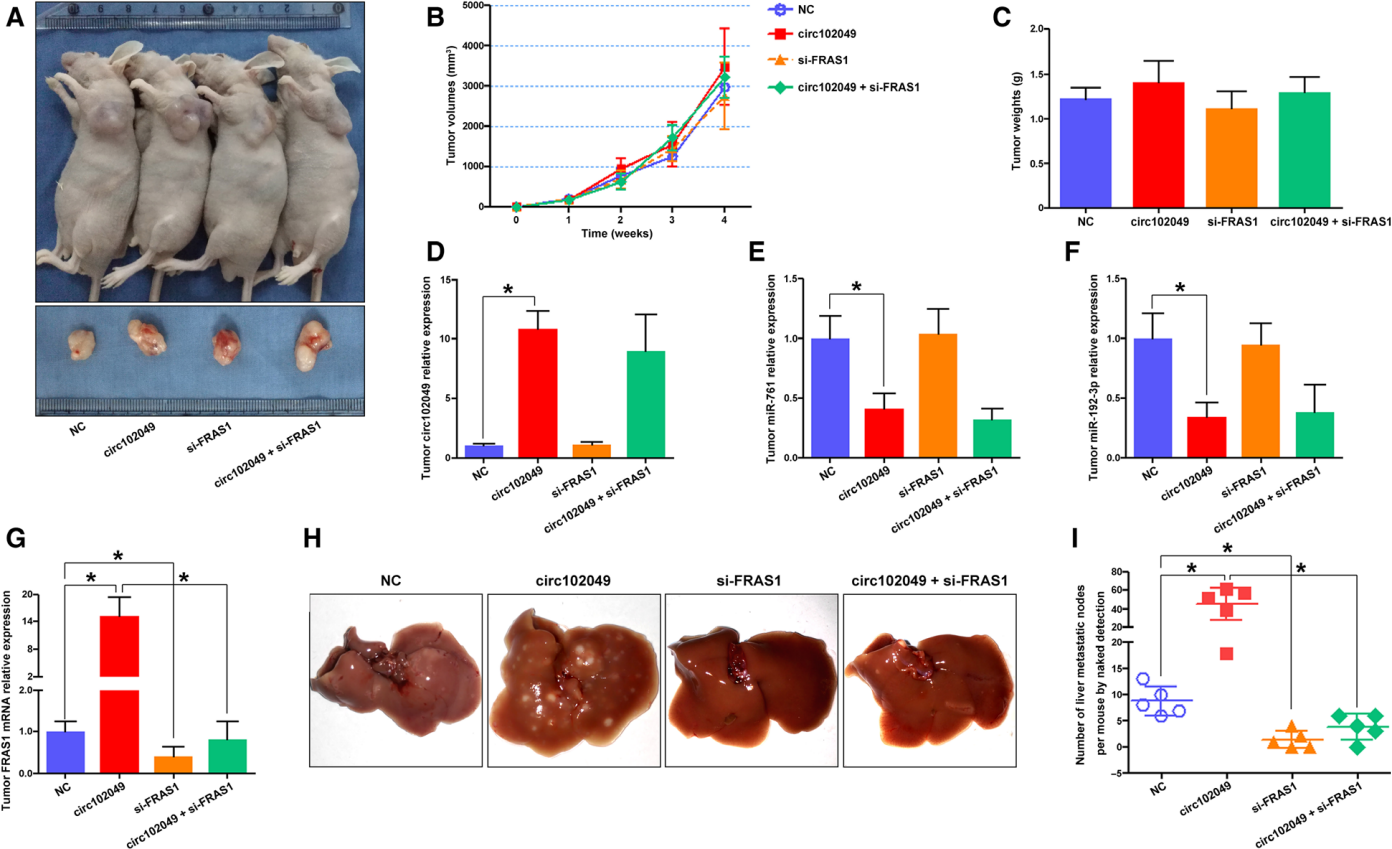
Circ102049 promoted colorectal liver metastasis *in vivo*. (A–C) Cells were injected subcutaneously into the flanks of BALB/c‐nude mice and TV was measured weekly. After 4 weeks, mice were sacrificed, and the tumor tissues were excised and weighed. (D–G) The regulatory relationships of circ102049, miR‐761, miR‐192‐3p and FRAS1 were confirmed in tumors by qRT‐PCR. (H,I) Cells were injected into the mice tail. After 1.5 months, the livers were excised and photographed. The liver metastatic nodes were then calculated (*P < 0.05; error bars represent standard deviation).

**Fig 8:**
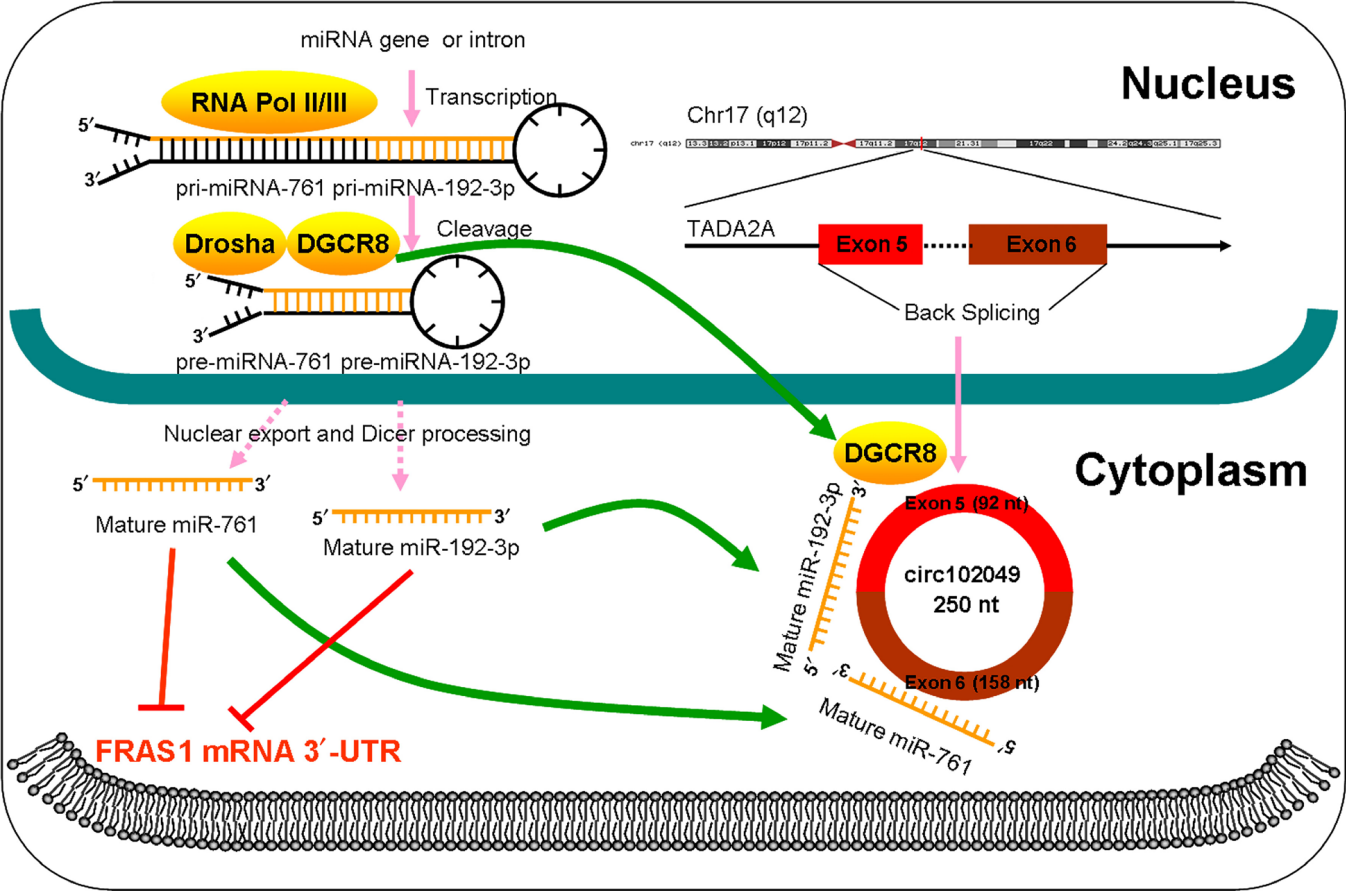
Schematic summary of circ102049 as a metastasis‐promoting ncRNA in CRC. Circ102049 promoted CRC development and progression via a miR‐761/miR‐192‐3p‐FRAS1‐dependent mechanism. Notably, due to the abnormal distribution of DGCR8 protein, circ102049 may also reduce the mature products of miR‐761 and miR‐192‐3p in cytoplasm indirectly.

Finally, we injected tumor cells subcutaneously into the flanks of BALB/c‐nude mice and investigated whether circ102049 promoted tumorigenesis *in vivo*. As expected *in vitro*, neither circ102049 or FRAS1 promoted tumor growth, and no rescued effects of si‐FRAS1 were observed in circ102049‐overexpressed mice (Fig. 7A–C). But, interestingly, the regulatory relationships of circ102049, miR‐761 (or miR‐192‐3p) and FRAS1 were confirmed in tumors by qRT‐PCR (Fig. 7D–G). To better observe the metastatic abilities *in vivo*, cells of different groups were injected into the mice tail. After 1.5 months, lung and liver metastases were observed. The data showed that neither circ102049 or FRAS1 expressions influenced the lung metastasis of SW1116 CRC cells (Fig. S8A,B). However, interestingly, circ102049 significantly promoted the liver metastasis of CRC cells, whereas silencing FRAS1 could weaken the metastatic capacity of tumor cells. More importantly, si‐FRAS1 also effectively antagonized the formation of liver metastases induced by circ102049 overexpression (Fig. 7H,I). Taken together, circ102049 may enhance colorectal liver metastasis specifically through a FRAS1‐dependent mechanism both *in vitro* and *in vivo* (Fig. 8).

## 4 Discussion

With the advances in high‐throughput sequencing and microarray technology, an increasing number of circRNA have been identified in human CRC carcinogenesis and metastasis. They are a subclass of noncoding RNA with high conservation and a very stable circular structure, making them ideal biomarkers for the diagnosis of disease. For instance, Yang *et al*. [25] compared six pairs of CRC tissues and their matched adjacent non‐tumor tissues using circRNA microarray and found that circPTK2 played a critical role in CRC growth and metastasis, and might serve as a promising biomarker for early diagnosis of metastasis. Similar circRNA such as circCAMSAP1, circ_001680 and ciRS‐122 were also proved to correlate significantly with advanced tumor stage, shortened OS and chemoresistance [26, 27, 28]. In the present study, we explored the circRNA microarray of CRC to identify new targets of colorectal liver metastasis. Circ102049 was the most up‐regulated circRNA in CRC primary tumors with and without liver metastasis. OS curves were plotted and the data showed that CRC patients with high circ102049 levels had a worse 5‐year OS. Analyzing the structure, we found that circ102049 was derived from the host gene *TADA2A* and had a stable loop structure based on head‐to‐tail splicing of two exons. Hence, we hypothesized that it could play a role in the development and progression of CRC. In functional experiments, circ102049 had no effects on the proliferation and apoptosis of CRC cells, but overexpression of circ102049 could significantly promote the adhesion, migration and invasion abilities *in vitro*. Opposing effects were also observed when circ102049 was silenced in CRC cells. These results indicated that circ102049 may play a metastasis‐promoting role in CRC.

To investigate further the underlying mechanism, we screened the downstream targets of circ102049 though bioinformatics analysis and qRT‐PCR. FRAS1 was identified as the potential target molecule. FRAS1 resides on chromosome 4q21.21 and encodes a putative extracellular matrix protein with a large extracellular domain, transmembrane region and a short C‐terminal cytoplasmic domain which appears to function in the regulation of epidermal‐basement membrane adhesion and organogenesis during development [29, 30]. Patients with Fraser syndrome, a genetic disorder that presents with congenital malformations such as cryptophthalmos, syndactylism and reno‐urinary anomalies, is caused by FRAS1 mutations [31]. In recent years, an increasing number of studies have identified FRAS1 that play crucial roles in tumor carcinogenesis. For example, a study by Xu *et al*. [32] identified serum FRAS1 as a human endometrial carcinoma‐derived protein in a xenograft model system. FRAS1 knockdown by shRNA could significantly reduce the migration and invasion ability of A549 cells through down‐regulation of FAK signaling in non‐small cell lung cancer [33]. Recently, Umeda *et al*. [34] found that FRAS1 contributed to the malignant phenotype of gastric cancer, especially liver metastasis. FRAS1 expression was associated with the activation of the EGFR and PI3K signaling pathways, and served as a predictive marker or a target for liver metastasis. To reveal the potential role of FRAS1 in CRC, we determined its mRNA expression in 202 CRC tissues. Similar to gastric cancer, our data demonstrated that the FRAS1 levels were significantly elevated in primary tumors and liver metastatic nodes of CRC patients. A series of rescue assays were employed and the *in vitro* results confirmed that silencing FRAS1 could effectively counteract the promotion of cell adhesion, migration and invasion induced by circ102049 overexpression. These findings strongly suggest that circ102049 promoted the metastasis abilities through a FRAS1‐dependent mechanism.

The functional mechanism of circRNA is diverse in human cancers, including acting as miRNA sponges, interacting with protein, regulating gene splicing or transcription, translating proteins or peptide, and epigenetic regulation [35, 36]. The results of RNA pull‐down and RIP assay implied that circ102049 could not interact with FRAS1 protein directly in CRC cells. More interestingly, using a circ102049‐specific probe, the CHIRP assay also confirmed that circ102049 could not be enriched on the FRAS1 promoter region indirectly through other proteins such as TF. These data negated direct combination or gene transcriptional regulation between these two molecules. In recent years, an increasing number of studies have reported that circRNA play crucial roles in CRC carcinogenesis by sponging miRNA [37, 38, 39]. Thus, a ceRNA mechanism between circ102049 and FRAS1 was highly speculative and was studied. First, a miRNA microarray was performed to analyze the differentially expressed miRNA. The Venn analysis concluded that only three miRNA (miR‐761, miR‐192‐3p and miR‐33a‐3p) may be involved. The results of PCR analysis in CRC tissues demonstrated that only the miR‐761 and miR‐192‐3p levels were significantly down‐regulated in CRC primary tumors and liver metastatic nodes. To confirm further our hypothesis of ceRNA, a series of experiments including the luciferase activity assay, FISH co‐localization by confocal scan imaging, anti‐Ago2 RIP assay and RNA pull‐down analysis all strongly confirmed the direct binding of circ102049 with miR‐761 or miR‐192‐3p in CRC cells. The downstream regulatory relationships of miR‐761 (or miR‐192‐3p) and FRAS1 were also discussed. Fortunately, the results of bioinformatics analysis, Western blot analysis and luciferase activity assay revealed that miR‐761 and miR‐192‐3p could specifically target FRAS1 3’‐UTR on 2271‐‐2277 and 1879‐‐1885 sites, respectively, which confirmed *FRAS1* as a common target gene of the circ102049‐miR‐761/miR‐192‐3p regulatory axis in CRC.

The RNA binding protein DGCR8, which is encoded by the DiGeorge syndrome critical region gene 8, is the minimal functional core of the nuclear microprocessor complex. It is essential for the biogenesis of canonical miRNA. During the miRNA maturing process, pri‐miRNA are first synthesized by RNA II polymerase in the nucleus. They are then converted into pre‐miRNA by a Drosha/DGCR8 microprocessor complex which is comprised of DROSHA, an RNase III superfamily member and its co‐factor DGCR8. Finally, the pre‐miRNA are exported to cytoplasm and converted into mature miRNA by DICER [40, 41, 42]. Lee *et al*. [43] reported that DICER1, DROSHA, DGCR8 and AGO2 were significantly dysregulated in clear cell renal cell carcinoma, suggesting that they were important in the pathophysiology of this malignancy. In 2014, Kim *et al*. [44] demonstrated for the first time that the DGCR8 mRNA expression was up‐regulated in CRC, which suggested its important role in the pathobiology of colorectal carcinogenesis. However, no further reports have especially emphasized the role of DGCR8 in CRC. In our study, we predicted one binding site for DGCR8 in the downstream region of the circ102049 mRNA transcript. The relationships between circ102049 and DGCR8 were then studied further. The RNA pull‐down assay and RIP analysis both indicated that DGCR8 protein could be combined with circ102049 directly in CRC cytoplasm. Moreover, circ102049 overexpression did not change the DGCR8 total mRNA or protein levels, whereas the nuclear and cytosolic DGCR8 proteins were abnormally distributed. In addition, our results showed that overexpression of DGCR8 could significantly up‐regulate mature miR‐761 and miR‐192‐3p levels in CRC cells. Considering the location of circ102049 and DGCR8 protein found in previous studies, we speculated that mature circ102049 in cytoplasm could effectively sponge DGCR8 proteins out of the nucleus, ultimately reducing the mature products of miR‐761 and miR‐192‐3p.

## 5 Conclusions

In summary, our findings suggest that circ102049 is highly expressed in primary CRC tumors with liver metastasis and is correlated with CRC patient prognosis. Mechanistically, circ102049 promoted CRC development and progression via a miR‐761/miR‐192‐3p‐FRAS1‐dependent mechanism. Notably, due to the abnormal distribution of DGCR8 protein in cells, circ102049 may indirectly reduce the mature products of miR‐761 and miR‐192‐3p in cytoplasm. The role of circ102049 in promoting colorectal liver metastasis was also confirmed in animal models. Our findings provide new clues that circ102049 might be a potential prognostic factor in CRC. The circ102049‐miR‐761/miR‐192‐3p–FRAS1 axis could be explored further as a therapeutic target for anti‐metastatic therapy in CRC patients with liver metastases.

## Conflict of interest

The authors declare no conflict of interest.

## Author contributions

LQ, YW and YX designed the study. QZ, DW, RR, ZX, XG, YH and FL conducted the experiments and data analysis. DW and XG provided technical support. QZ and DW wrote the manuscript. All authors read and approved the final manuscript.

### Peer Review

The peer review history for this article is available at https://publons.com/publon/10.1002/1878‐0261.12840.

## Supporting information


**Fig. S1.** Silencing circ102049 suppressed the adhesion, migration and invasion of CRC cells. (A) circ102049 expression was effectively silenced by siRNA using qRT‐PCR. (B,C) The proliferative ability was determined by the CCK‐8 assay. (D,E) The colony formation assay was performed and calculated. (F,G) The apoptotic rates of CRC cells were determined through Annexin‐V staining. (H) The cell adhesion ability. (I–L) Cell migration and invasion ability (*P < 0.05; error bars represent standard deviation).

**Fig. S2.** A series of rescue assays were employed. si‐FRAS1 treatment could counteract the circ102049 overexpression‐mediated promotions on cell adhesion, migration and invasion in CRC cells. (A) mRNA expression of FRAS1 was determined after si‐FRAS1 transfection in CRC cells. (B,C) The proliferative ability was determined by CCK‐8 assay. (D,E) The colony formation assay was performed and calculated. (F,G) The apoptotic rates of CRC cells were determined through Annexin‐V staining. (H) The ability of cell adhesion was determined. (I,J) The ability of cell migration and invasion was determined; Scale bar = 10 μm (*P < 0.05; error bars represent standard deviation).

**Fig. S3.** Circ102049 could not bind to FRAS1 protein directly or transcriptionally activate FRAS1 gene in CRC cells. (A,B) mRNA or protein expressions of FRAS1 were determined after si‐circ102049 (Junction 1) treatment by qRT‐PCR or Western blot. (C,D) RNA pull‐down and RIP assay suggested that there were no direct interactions between circ102049 and FRAS1. (E) Using a circ102049‐specific probe, the ChIRP assay confirmed that circ102049 could not be enriched on FRAS1 promoter region (−2000 to 0 bp) (*P < 0.05; error bars represent standard deviation).

**Fig. S4.** Circ102049 sponged miR‐761 and miR‐192‐3p in CRC cells. (A) The potential binding sites between circ102049 and miR‐761 (or miR‐192‐3p) were predicted. (B,C) The p‐luc‐circ102049‐wild‐ or mutant type reporter vectors were constructed and the luciferase activities were determined when miR‐761 (or miR‐192‐3p) mimics were co‐transfected into SW620 cells. (D) The FISH results showed that circ102049 and miR‐761 (or miR‐192‐3p) were preferentially co‐localized in the cytoplasm of SW620 cells. Scale bar = 10 μm. (E,F) The results of RIP assay showed that endogenous circ102049 pull‐down by AGO2 was specifically enriched in SW620 cells upon overexpression of miR‐761 or miR‐192‐3p. (G–I) RNA pull‐down analysis demonstrated that endogenous miR‐761 or miR‐192‐3p could also be significantly pulled down by biotinylated probes against circ102049 (*P < 0.05; error bars represent standard deviation).

**Fig. S5.** *FRAS1* was a common target gene of miR‐761 and miR‐192‐3p in CRC cells. (A) The binding sites between miR‐761 (or miR‐192‐3p) and *FRAS1* were predicted by Targetscan. (B,C) Western blot analysis showed the expression of FRAS1 protein after transfection of miR‐761 (or miR‐192‐3p) mimics or inhibitors. (D,E) The results of luciferase reporter assay demonstrated that miR‐761 and miR‐192‐3p could specifically target FRAS1 3’‐UTR on the sites of 2271–2277 and 1879–1885, respectively (*P < 0.05; error bars represent standard deviation).

**Fig. S6.** Pearson’s correlations show relationships of circ102049, miR‐761, miR‐192‐3p and FRAS1 in 202 CRC tissues. (A) The positive relationships between circ102049 and FRAS1. (B,C) circ102049 was negatively correlated with miR‐761 (or miR‐192‐3p). (D,E) FRAS1 was negatively correlated with miR‐761 (or miR‐192‐3p) (*P < 0.05; error bars represent standard deviation).

**Fig. S7.** DGCR8 mRNA expressions in 202 CRC patients with (*n* = 65) and without liver metastasis (*n* = 137) were determined by qRT‐PCR (*P < 0.05).

**Fig. S8.** The circ102049 did not increase colorectal lung metastasis *in vivo*. (A) Cells were injected into the mice tail. After 1.5 months, the lungs were excised and photographed. (B) Number of the lung metastatic nodes (*P < 0.05; error bars represent standard deviation).

**Table S1.** Primers used for circRNA and gene expression analysis.

**Table S2.** Sequences of siRNA, miRNA mimics or inhibitors.

**Table S3.** Primer sequences targeting FRAS1 promoter in ChIRP assay.

**Table S4.** General information on CRC cell lines from ATCC.

**Table S5.** Univariate and multivariate analysis of circ102049 survival in 202 patients with CRC.
