# Supplementary figures and images for: Circular RNA profiling identifies circ102049 as a key regulator of colorectal liver metastasis
Source: Mol Oncol. 2020 Dec 29;15(2):623–41. doi: 10.1002/1878-0261.12840 (PMC7858140; doi:10.1002/1878-0261.12840)

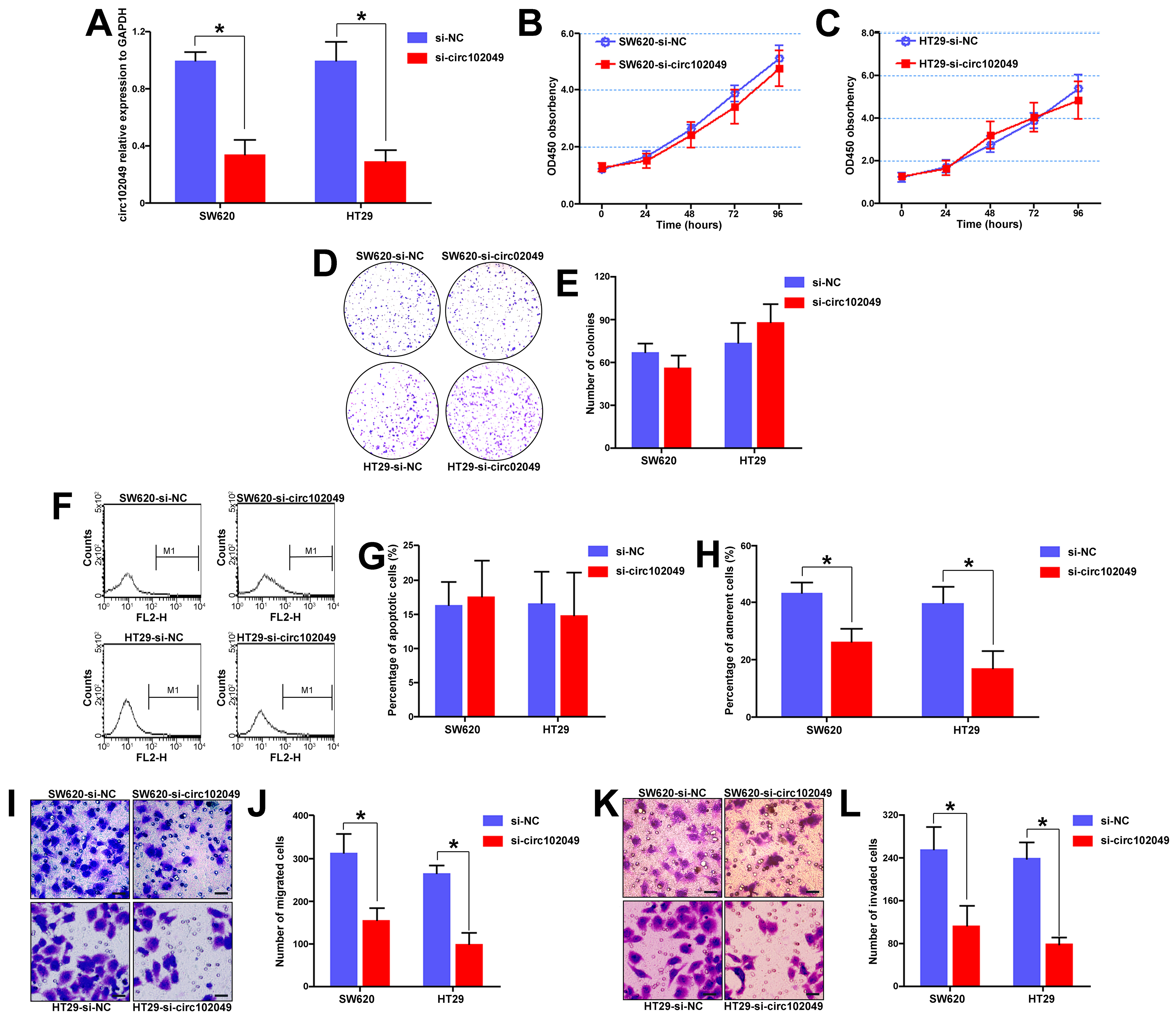

Supplement: Supplementary file 1 — Fig. S1. Silencing circ102049 suppressed the adhesion, migration and invasion of CRC cells. (A) circ102049 expression was effectively silenced by siRNA using qRT‐PCR. (B,C) The proliferative ability was determined by the CCK‐8 assay. (D,E) The colony formation assay was performed and calculated. (F,G) The apoptotic rates of CRC cells were determined through Annexin‐V staining. (H) The cell adhesion ability. (I–L) Cell migration and invasion ability (*P < 0.05; error bars represent standard deviation). [file MOL2-15-623-s001.tif]

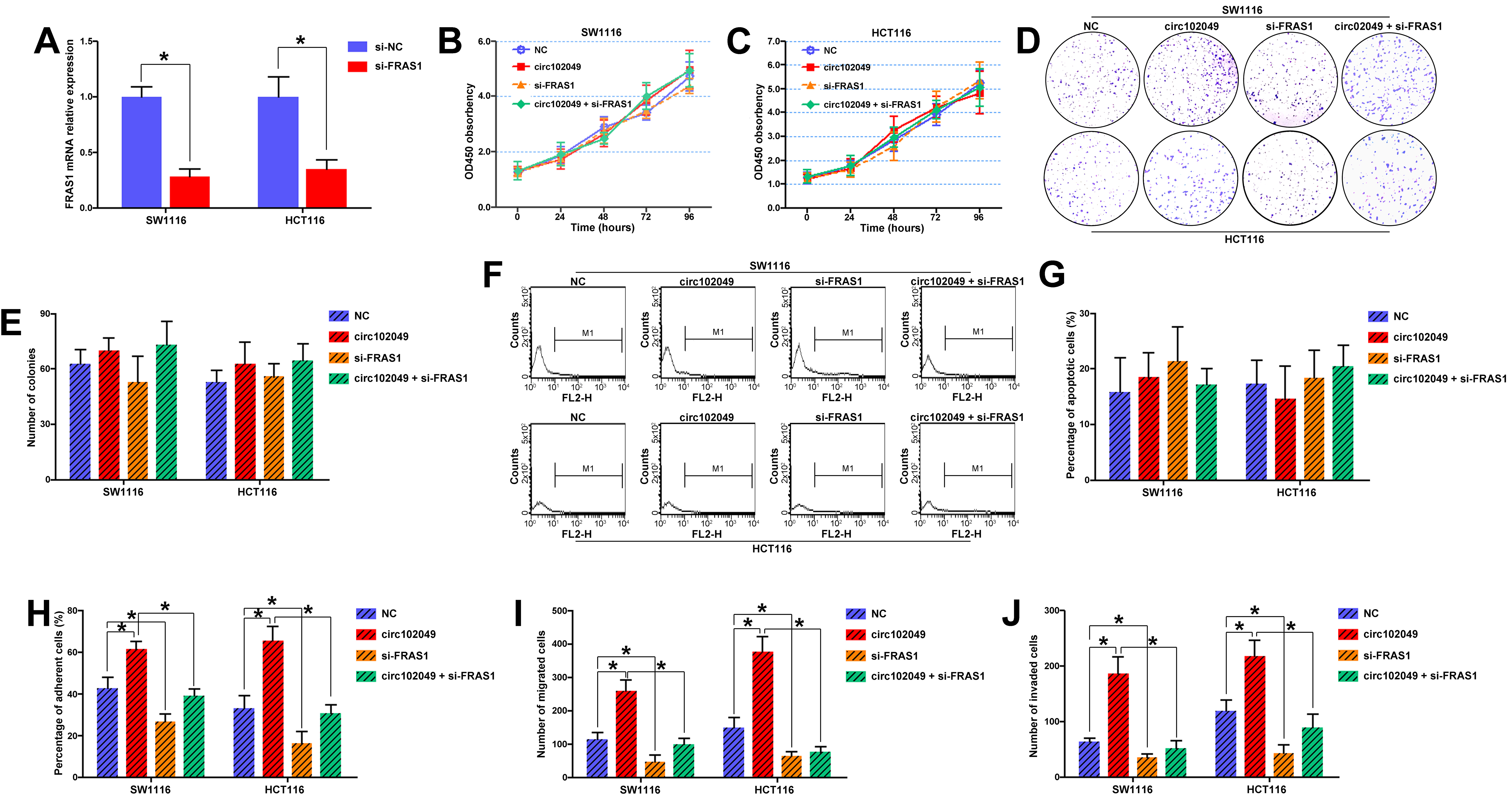

Supplement: Supplementary file 2 — Fig. S2. A series of rescue assays were employed. si‐FRAS1 treatment could counteract the circ102049 overexpression‐mediated promotions on cell adhesion, migration and invasion in CRC cells. (A) mRNA expression of FRAS1 was determined after si‐FRAS1 transfection in CRC cells. (B,C) The proliferative ability was determined by CCK‐8 assay. (D,E) The colony formation assay was performed and calculated. (F,G) The apoptotic rates of CRC cells were determined through Annexin‐V staining. (H) The ability of cell adhesion was determined. (I,J) The ability of cell migration and invasion was determined; Scale bar = 10 μm (*P < 0.05; error bars represent standard deviation). [file MOL2-15-623-s002.tif]

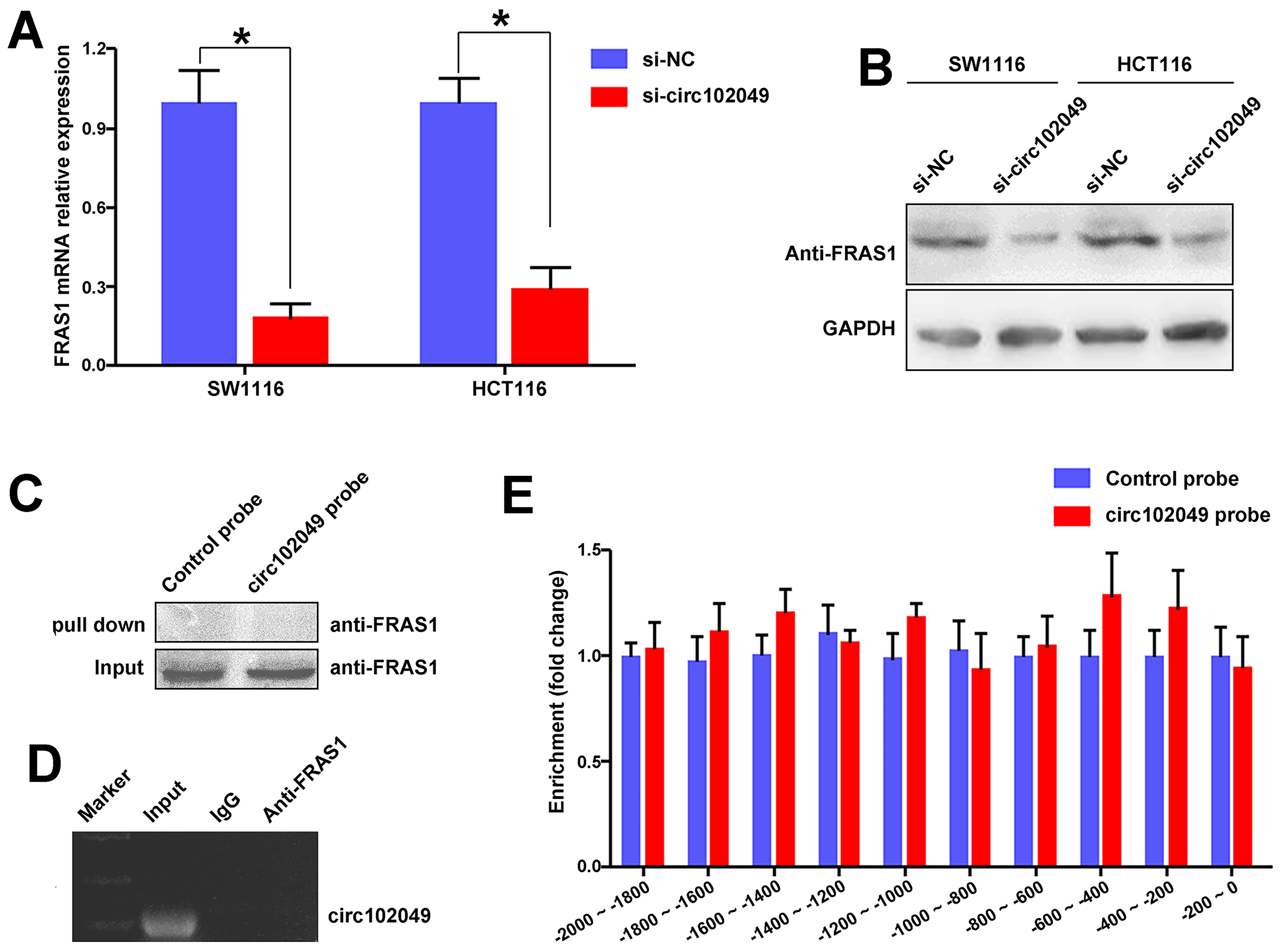

Supplement: Supplementary file 3 — Fig. S3. Circ102049 could not bind to FRAS1 protein directly or transcriptionally activate FRAS1 gene in CRC cells. (A,B) mRNA or protein expressions of FRAS1 were determined after si‐circ102049 (Junction 1) treatment by qRT‐PCR or Western blot. (C,D) RNA pull‐down and RIP assay suggested that there were no direct interactions between circ102049 and FRAS1. (E) Using a circ102049‐specific probe, the ChIRP assay confirmed that circ102049 could not be enriched on FRAS1 promoter region (−2000 to 0 bp) (*P < 0.05; error bars represent standard deviation). [file MOL2-15-623-s003.tif]

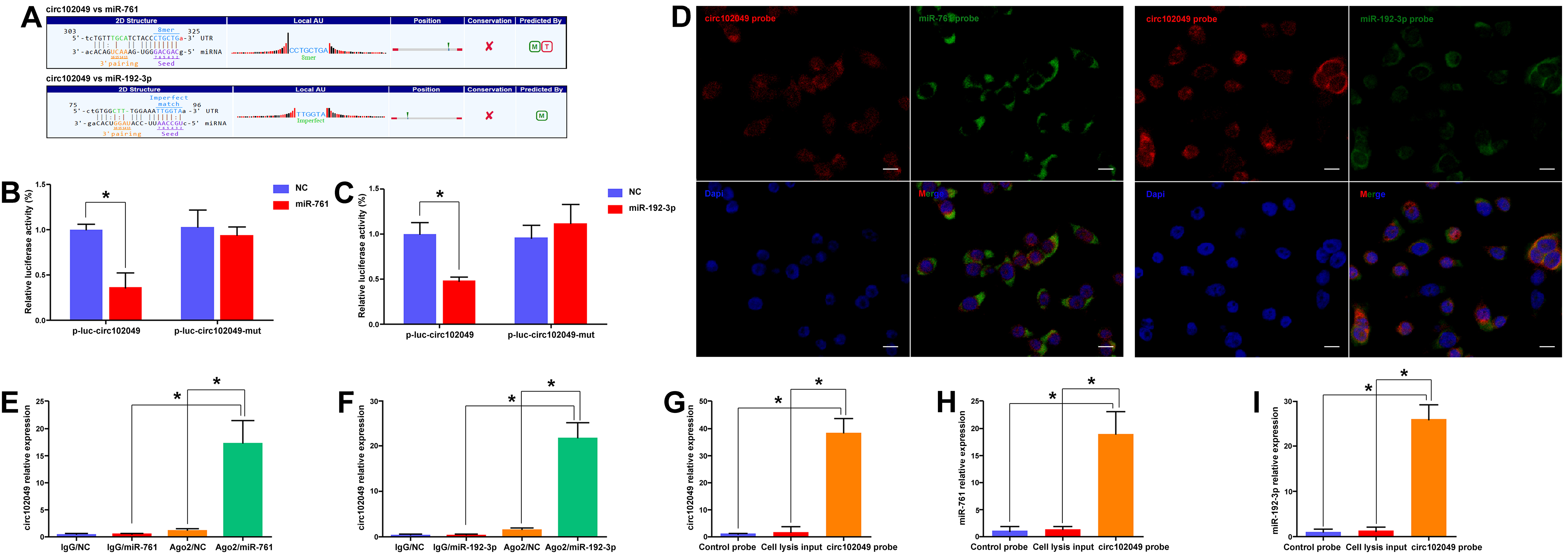

Supplement: Supplementary file 4 — Fig. S4. Circ102049 sponged miR‐761 and miR‐192‐3p in CRC cells. (A) The potential binding sites between circ102049 and miR‐761 (or miR‐192‐3p) were predicted. (B,C) The p‐luc‐circ102049‐wild‐ or mutant type reporter vectors were constructed and the luciferase activities were determined when miR‐761 (or miR‐192‐3p) mimics were co‐transfected into SW620 cells. (D) The FISH results showed that circ102049 and miR‐761 (or miR‐192‐3p) were preferentially co‐localized in the cytoplasm of SW620 cells. Scale bar = 10 μm. (E,F) The results of RIP assay showed that endogenous circ102049 pull‐down by AGO2 was specifically enriched in SW620 cells upon overexpression of miR‐761 or miR‐192‐3p. (G–I) RNA pull‐down analysis demonstrated that endogenous miR‐761 or miR‐192‐3p could also be significantly pulled down by biotinylated probes against circ102049 (*P < 0.05; error bars represent standard deviation). [file MOL2-15-623-s004.tif]

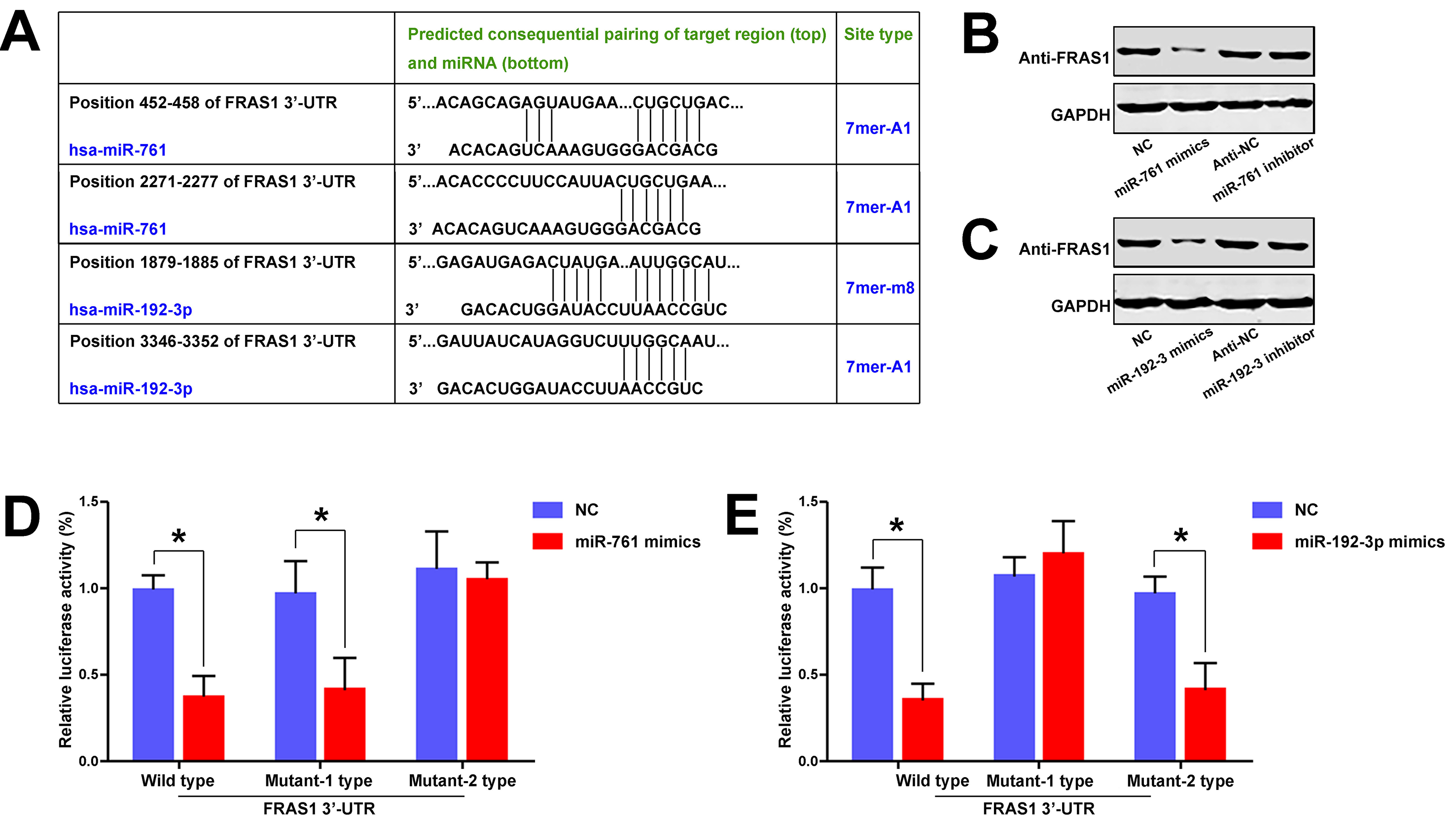

Supplement: Supplementary file 5 — Fig. S5. FRAS1 was a common target gene of miR‐761 and miR‐192‐3p in CRC cells. (A) The binding sites between miR‐761 (or miR‐192‐3p) and FRAS1 were predicted by Targetscan. (B,C) Western blot analysis showed the expression of FRAS1 protein after transfection of miR‐761 (or miR‐192‐3p) mimics or inhibitors. (D,E) The results of luciferase reporter assay demonstrated that miR‐761 and miR‐192‐3p could specifically target FRAS1 3’‐UTR on the sites of 2271–2277 and 1879–1885, respectively (*P < 0.05; error bars represent standard deviation). [file MOL2-15-623-s005.tif]

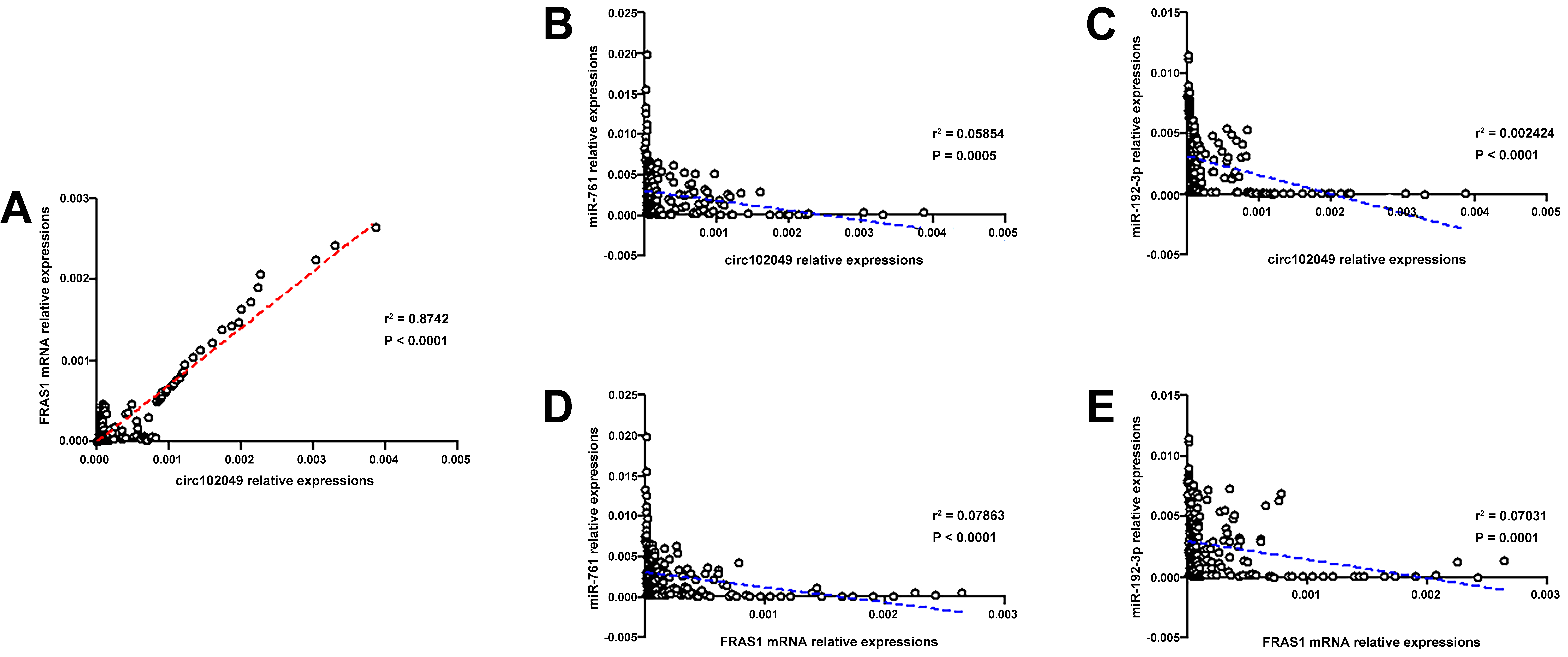

Supplement: Supplementary file 6 — Fig. S6. Pearson’s correlations show relationships of circ102049, miR‐761, miR‐192‐3p and FRAS1 in 202 CRC tissues. (A) The positive relationships between circ102049 and FRAS1. (B,C) circ102049 was negatively correlated with miR‐761 (or miR‐192‐3p). (D,E) FRAS1 was negatively correlated with miR‐761 (or miR‐192‐3p) (*P < 0.05; error bars represent standard deviation). [file MOL2-15-623-s006.tif]

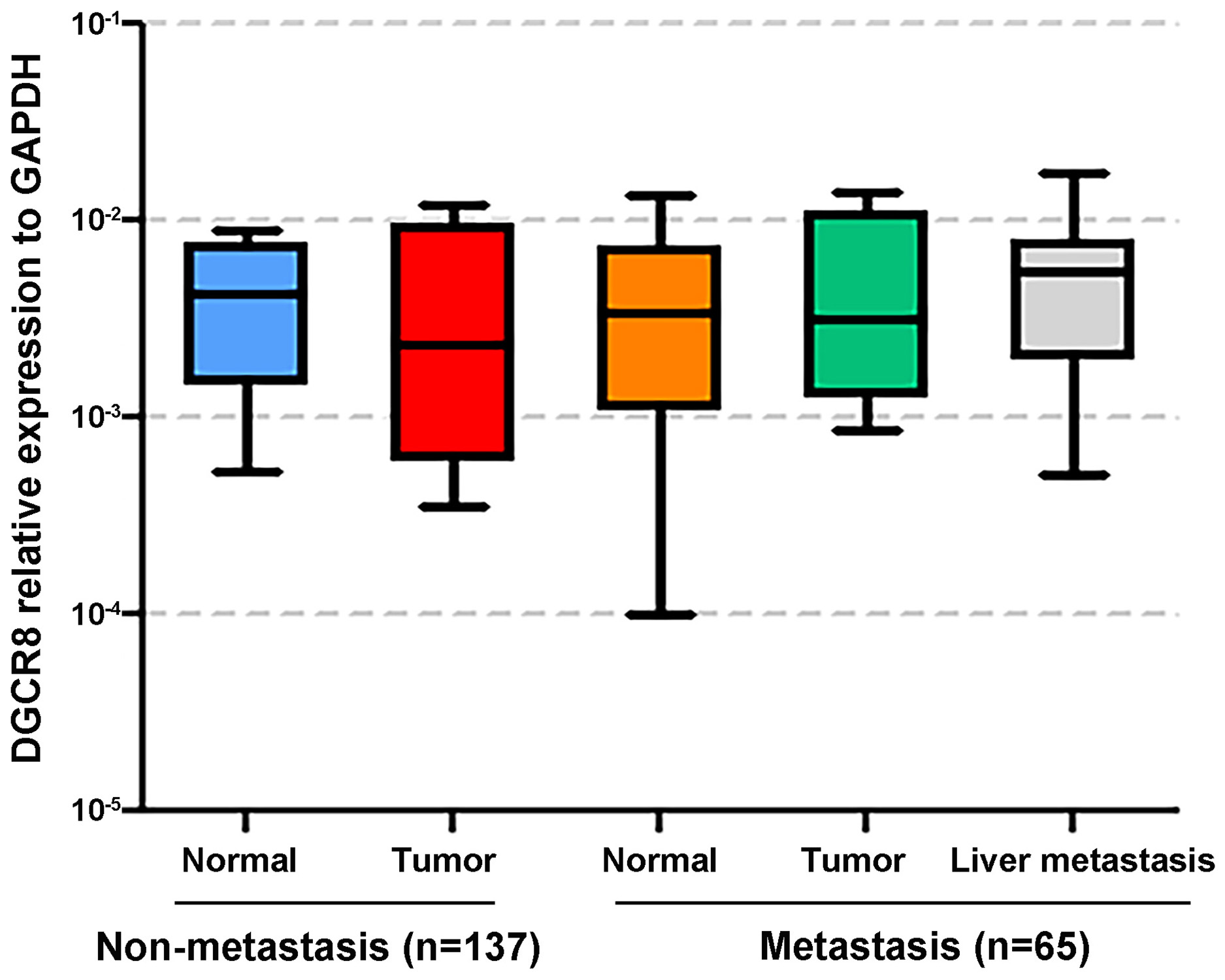

Supplement: Supplementary file 7 — Fig. S7. DGCR8 mRNA expressions in 202 CRC patients with (n = 65) and without liver metastasis (n = 137) were determined by qRT‐PCR (*P < 0.05). [file MOL2-15-623-s007.tif]

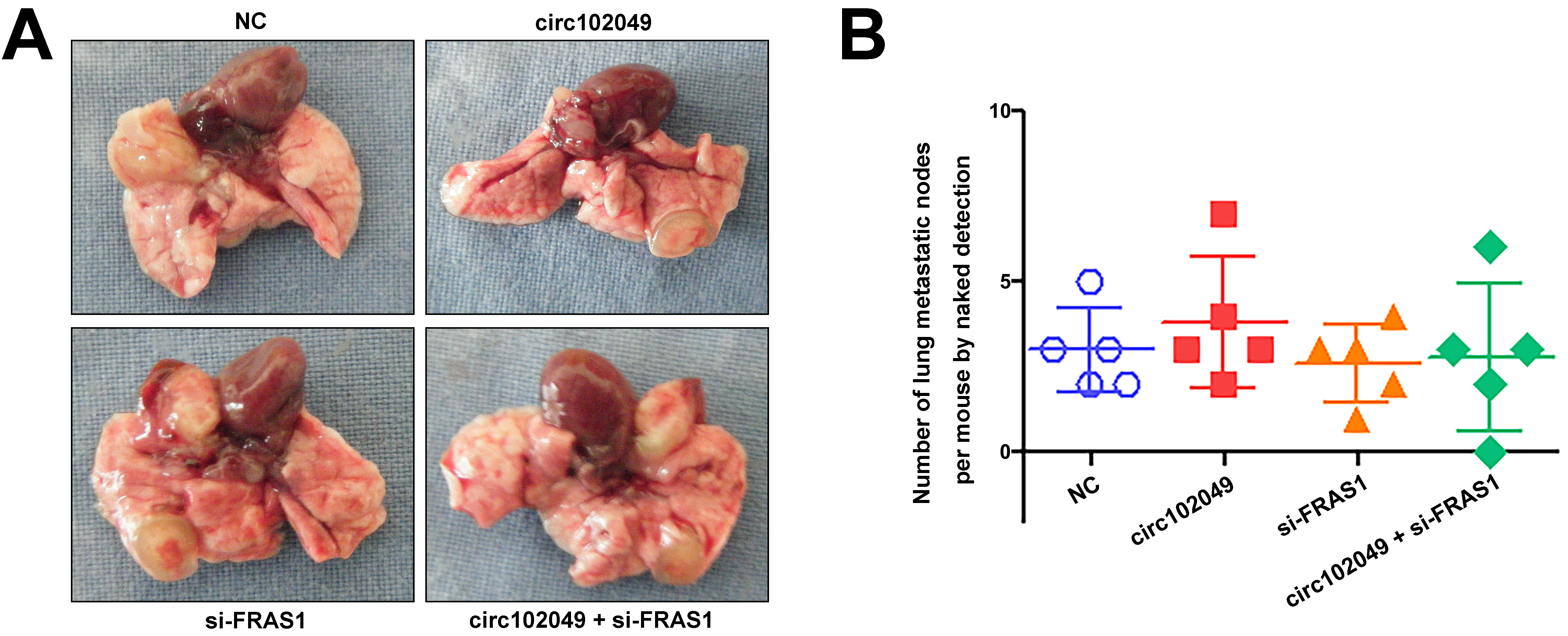

Supplement: Supplementary file 8 — Fig. S8. The circ102049 did not increase colorectal lung metastasis in vivo. (A) Cells were injected into the mice tail. After 1.5 months, the lungs were excised and photographed. (B) Number of the lung metastatic nodes (*P < 0.05; error bars represent standard deviation). [file MOL2-15-623-s008.tif]
