# Supplementary material for: Circular RNA profiling identifies circ102049 as a key regulator of colorectal liver metastasis
Source: Mol Oncol. 2020 Dec 29;15(2):623–41. doi: 10.1002/1878-0261.12840 (PMC7858140; doi:10.1002/1878-0261.12840)
Supplement: Supplementary file 9 — Table S1. Primers used for circRNA and gene expression analysis. [file MOL2-15-623-s009.doc]

**Table S1:** Primers used for circRNAs and gene expression analysis

| **Gene name** | **Sense** | **Anti-sense** | **Product size** |
| --- | --- | --- | --- |
| Linear 102049 (Primer1, Convergent) | AGAGACGGGGTTTCACTGTGTTAG | GCTGAGGCAGGAGAATCACTTG | 231 |
| Linear 102049 (Primer 2) | AAAGTGCTGGGAGTATAGGCGT | CCAAAGCCACAGTCCATCACA | 177 |
| Circ102049 (Primer 3) | TGATGGACTGTGGCTTTGG | TCAGGTTCAGCAGGGTAGATG | 135 |
| Linear 102049 (Primer 4) | TGAAACAAGCAGAGGAAGCAAA | GCCATACTAACTCCTCCTATCTTCC | 181 |
| Circ102049 (Primer 5, Divergent) | GAAACAAGCAGAGGAAGCAAAA | CAAAGCCACAGTCCATCACA | 135 |
| Linear 102049 (Primer 6) | GTTAGGAGGTGCAGTGACCC | ACAAGCCCATCTGTCCCAAG | 243 |
| TSPAN10 | AACACCTGCCTGTTACGTGG | TCTGGGTCGTCCTGGTAGTG | 158 |
| FN1 | CGGTGGCTGTCAGTCAAAG | AAACCTCGGCTTCCTCCATAA | 130 |
| MMP24 | GCCGGGCAGAACTGGTTAAA | CCCGTAAAACTGCTGCATAGT | 120 |
| SLAMF9 | TCTGGTCCTCTCACAAAAGTCT | CCTGGTAGTGTGGATTGGTCA | 90 |
| SCUBE3 | CAGAACACCCCGAGGTCATAC | GCCAGGGATGTTGACACAGTC | 135 |
| CD36 | CTTTGGCTTAATGAGACTGGGAC | GCAACAAACATCACCACACCA | 134 |
| FGFR1 | CCCGTAGCTCCATATTGGACA | TTTGCCATTTTTCAACCAGCG | 138 |
| ALPL | AACATCAGGGACATTGACGTG | GTATCTCGGTTTGAAGCTCTTCC | 159 |
| NRP1 | GGCGCTTTTCGCAACGATAAA | TCGCATTTTTCACTTGGGTGAT | 107 |
| KIF26B | GCTGGGAATAAAGAGAGGCTTG | ACTCCTCGTATGCTTTCCGGT | 130 |
| ANGPTL4 | GTCCACCGACCTCCCGTTA | CCTCATGGTCTAGGTGCTTGT | 212 |
| LAMA5 | CCCACCGAGGACCTTTACTG | GGTGTGCCTTGTTGCTGTT | 121 |
| FLRT3 | GATGAATTTCCTACCAACCTCCC | TGTCTCGGAATGCTCCCTCTT | 172 |
| BCAM | CAGGTCACAATGCACGACAC | CACCACGCACACGTAGTCT | 117 |
| ADAM8 | CAGACCTGCACCTGATCGAG | CATTGTCCACGACCACATACA | 228 |
| ADAMTSL2 | CTTCAACGGCAACTACAAGGT | TCGATTCCGGTCTCATAGACAT | 102 |
| PCDHA8 | CGCCAGTGTTCCGGGTAAAA | CACGGAGTTAGCTCCAACATC | 113 |
| BAMBI | ATGCTCTCCCGTTTGCACTAC | AGGATCTTATCGTTGCTGAGGT | 161 |
| FRAS1 | AATAGCTGCCAACCAATGCTG | CAAGAGCACACACTACATGGAG | 123 |
| PXDN | AGGATACCTAGTGGAGCATTTGA | CGAATCTGGGTCCAAAGTTTCTA | 162 |
| LYPD3 | GGTCGTGAGCTGCTACAAC | GGTCAGAGTTACAGCGGGAC | 203 |
| COL13A1 | GAAAAGGGACCTCGCGGTAA | AACCTCTGCTCCTGGATTGC | 207 |
| SRPX | TGCTCCAGCGACGGTGATA | TGTGGACACAATGAGGAGTCT | 219 |
| TGFB3 | AACGGTGATGACCCACGTC | CCGACTCGGTGTTTTCCTGG | 119 |
| RASSF8 | AGTTCAGAGGATTGTTTGTGGAG | CACATCACTAGCATACTGCCC | 193 |
| DGCR8 | AGCGTGAGCTTTACCGAGAG | CTACCCCGTCACCAACACTC | 157 |
| GAPDH | TGTTGCCATCAATGACCCCTT | CTCCACGACGTACTCAGCG | 202 |
