# Supplementary material for: Circular RNA profiling identifies circ102049 as a key regulator of colorectal liver metastasis
Source: Mol Oncol. 2020 Dec 29;15(2):623–41. doi: 10.1002/1878-0261.12840 (PMC7858140; doi:10.1002/1878-0261.12840)
Supplement: Supplementary file 10 — Table S2. Sequences of siRNA, miRNA mimics or inhibitors. [file MOL2-15-623-s010.doc]

**Table S2:** The sequences of siRNAs, miRNA mimics or inhibitors

| **siRNA name** | **Sense strand** | **Anti-sense stand** |
| --- | --- | --- |
| si-line 102049 | GCUUGAUUAUGGUGAAUAU | CGAACUAAUACCACUUAUA |
| si-circ102049 (Exon 2) | GAGUGUGAGAAGCACUAUA | CUCACACUCUUCGUGAUAU |
| si-circ102049 (Junction 1) | CAUCACUUUACCUUACCGA | GUAGUGAAAUGGAAUGGCU |
| si-circ102049 (Junction 2) | CUUUGGAAAUUGGCAGGAU | GAAACCUUUAACCGUCCUA |
| si-FRAS1 | CCCGGGAUGAAGAAUUAAU | GGGCCCUACUUCUUAAUUA |
| mimics NC | UUCUCCGAACGUGUCACGUTT | ACGUGACACGUUCGGAGAATT |
| inhibitor NC | CAGUACUUUUGUGUAGUACAA |  |
| miR-761 mimics | GCAGCAGGGUGAAACUGACACA | UGUCAGUUUCACCCUGCUGCUU |
| miR-761 inhibitor | UGUGUCAGUUUCACCCUGCUGC |  |
| miR-192-3p mimics | CUGCCAAUUCCAUAGGUCACAG | GUGACCUAUGGAAUUGGCAGUU |
| miR-192-3p inhibitor | CUGUGACCUAUGGAAUUGGCAG |  |
