# Supplementary material for: Circular RNA profiling identifies circ102049 as a key regulator of colorectal liver metastasis
Source: Mol Oncol. 2020 Dec 29;15(2):623–41. doi: 10.1002/1878-0261.12840 (PMC7858140; doi:10.1002/1878-0261.12840)
Supplement: Supplementary file 11 — Table S3. Primer sequences targeting FRAS1 promoter in ChIRP assay. [file MOL2-15-623-s011.doc]

**Table S3：**Primer sequences targeting FRAS1 promoter in ChIRP assay

| **Region** | **Sense** | **Anti-sense** |
| --- | --- | --- |
| -2000~-1800 | 5’-GAGTGTGTTGGAAGCTGAGT-3’ | 5’-AGAATCACTTGAACCCGGGA-3’ |
| -1800~-1600 | 5’-CAAGTGATTCTCGTGCCTCAG-3’ | 5’-GCCTGTAATCCCAGCACTTT-3’ |
| -1600~-1400 | 5’-TTTGGGAGGAGGTTAAGGCC-3’ | 5’-ACATTTCGCCCTCAGTCTCT-3’ |
| -1400~-1200 | 5’-TCAGAGCCAGGTTGTCAGAG-3’ | 5’-GGTCCCACTTGTTTCCAGTC-3’ |
| -1200~-1000 | 5’-GGAAACAAGTGGGACCCC-3’ | 5’-TCGTTTTATAAGGGAACAGTTGC-3’ |
| -1000~-800 | 5’-TGCCGTCAGTCAGTCCTG-3’ | 5’-ATGCACCTTTCCCTTTCCCT-3’ |
| -800~-600 | 5’-CCAGAGCGAGGAAGAAGGAT-3’ | 5’-GAAGGCAGATGAACCGTGTC-3’ |
| -600~-400 | 5’-GACACGGTTCATCTGCCTTC-3’ | 5’-GAAAGGGAGAAGCGAGGAGG-3’ |
| -400~-200 | 5’-CCCACGTGACTCTCTGGAAT-3’ | 5’-CCAAACTCAAGGCCACTCTG-3’ |
| -200~0 | 5’-TCCGTGCAATTAAAATTATGGCT-3’ | 5’-TGCTACTTTCTCGTCCCCG-3’ |
