# Supplementary material for: Circular RNA profiling identifies circ102049 as a key regulator of colorectal liver metastasis
Source: Mol Oncol. 2020 Dec 29;15(2):623–41. doi: 10.1002/1878-0261.12840 (PMC7858140; doi:10.1002/1878-0261.12840)
Supplement: Supplementary file 12 — Table S4. General information on CRC cell lines from ATCC. [file MOL2-15-623-s012.doc]

**Table S4:** The general information of CRC cell lines from ATCC

| **Cell Line** | **General information (Tissue)** | **General information (Disease)** |
| --- | --- | --- |
| SW1116 | Colon | Dukes' type A, grade III, colorectal adenocarcinoma |
| SW620 | Colon; derived from metastatic site: lymph node | Dukes' type C, colorectal adenocarcinoma |
| HCT116 | Colon | Colorectal carcinoma |
| DLD-1 | Colon | Dukes' type C, colorectal adenocarcinoma |
| KM12 | - | - |
| HT29 | Colon | Colorectal adenocarcinoma |
| LOVO | Colon; derived from metastatic site: left supraclavicular region | Dukes' type C, grade IV, colorectal adenocarcinoma |
