# Supplementary material for: Circular RNA profiling identifies circ102049 as a key regulator of colorectal liver metastasis
Source: Mol Oncol. 2020 Dec 29;15(2):623–41. doi: 10.1002/1878-0261.12840 (PMC7858140; doi:10.1002/1878-0261.12840)
Supplement: Supplementary file 13 — Table S5. Univariate and multivariate analysis of circ102049 survival in 202 patients with CRC. [file MOL2-15-623-s013.doc]

Table S5: Univariate and multivariate analysis of circ102049 survival in 202 patients with CRC

| **Clinical Variables** | **Univariate analysis** | | | P Value | **Multivariate analysis** | | | P Value |
| --- | --- | --- | --- | --- | --- | --- | --- | --- |
|  | HR | 95%CI | |  | HR | 95%CI | |  |
| Gender (Male vs Female) | 0.981 | 0.646 | 1.492 | 0.93 |  |  |  |  |
| Age (≥60 vs <60 years old) | 1.152 | 0.753 | 1.763 | 0.513 |  |  |  |  |
| Size (≥5 vs <5 cm) | 1.181 | 0.778 | 1.794 | 0.435 |  |  |  |  |
| LNM (Postive vs Negative) | 1.676 | 1.085 | 2.589 | 0.02* | 1.302 | 0.829 | 2.045 | 0.252 |
| TNM stage (I/II vs III/IV) | 1.038 | 0.681 | 1.583 | 0.861 |  |  |  |  |
| circ102049 (High vs Low expression) | 2.057 | 1.331 | 3.178 | 0.001* | 2.021 | 1.292 | 3.161 | 0.002* |
| FRAS1 (High vs Low expression) | 1.87 | 1.176 | 2.974 | 0.008* | 1.87 | 1.166 | 3 | 0.009* |
| miR-192-3p (High vs Low expression) | 1.053 | 0.691 | 1.606 | 0.81 |  |  |  |  |
| miR-761 (High vs Low expression) | 1.084 | 0.714 | 1.647 | 0.705 |  |  |  |  |

LNM: Lymph node metastasis; HR: Hazard ratio; CI: Confidence interval; *P<0.05
